# A mechanism for the disrupted redox regulation of vascular contractility during aging

**DOI:** 10.1016/j.isci.2025.114264

**Published:** 2025-11-27

**Authors:** Leonardo Y. Tanaka, Lucas F. Gutierre, Ricardo C. Massucatto, Geovana S. Garcia, Carolina M. Portas, Victor Debbas, Júlia M.F. de Souza, Tiphany C. De Bessa, Lívia Teixeira, Percíllia V.S. Oliveira, Beatriz P. Souza, Samantha K. Teixeira, Paola C. Branco, Ayumi A. Miyakawa, Renato S. Gaspar, Daniela Kajihara, Iuri C. Valadão, Amit Bhowmik, Kate Carroll, Francisco R.M. Laurindo

**Affiliations:** 1 Laboratório de Biologia Vascular, Instituto do Coração (InCor), Hospital das Clínicas HCFMUSP, Faculdade de Medicina, Universidade de São Paulo, São Paulo, SP, Brazil; 2 Departamento de Medicina Translacional, Faculdade de Ciências Médicas, Universidade Estadual de Campinas, Campinas, SP, Brazil; 3 Laboratório de Genética e Cardiologia Molecular, Instituto do Coração (InCor), Hospital das Clínicas HCFMUSP, Faculdade de Medicina, Universidade de São Paulo, São Paulo, SP, Brazil; 4 Department of Chemistry, The Scripps Research Institute, Jupiter, FL 33458, USA

**Keywords:** Vascular anatomy, Age, Biochemistry, Cell biology

## Abstract

Vascular dysfunction contributes to aging-related phenotype, but mechanisms remain unclear. We propose that aging promotes a deregulated convergence between cellular redox processes and mechanoregulation. We focus on Protein Disulfide Isomerase-A1 (PDI), an endoplasmic reticulum redox chaperone known to modulate NADPH oxidase complexes and to fine-tune cytoskeletal remodeling. Our hypothesis is that PDI connects oxidant generation to actin cytoskeleton remodeling via the modulation of protein sulfenylation, an oxidative post-translational modification. We first show that protein sulfenylation supports vascular contractility and F-actin assembly during mechanoadaptation or agonist-induced contraction. Meanwhile, PDI supports sulfenylation-dependent actin remodeling. Moreover, aged murine arteries lose the sulfenic acid-related component of contractility, while PDI overexpression over-rides this dysfunction and restores aging-related vascular contractility. We further confirm a direct PDI-actin interaction modulated by sulfenic acid. Overall, signaling connections between PDI and sulfenylated proteins behave as an upstream integrative system regulating F-actin assembly, a mechanism that is impaired during aging-induced vascular dysfunction.

## Highlights

• Actin reorganization promotes the PDI-dependent local production of hydrogen peroxide
• PDI links oxidant generation to protein sulfenylation, supporting vasoconstriction
• PDI overexpression prevents aging-associated contractile dysfunction

## Graphical abstract

**Figure:**
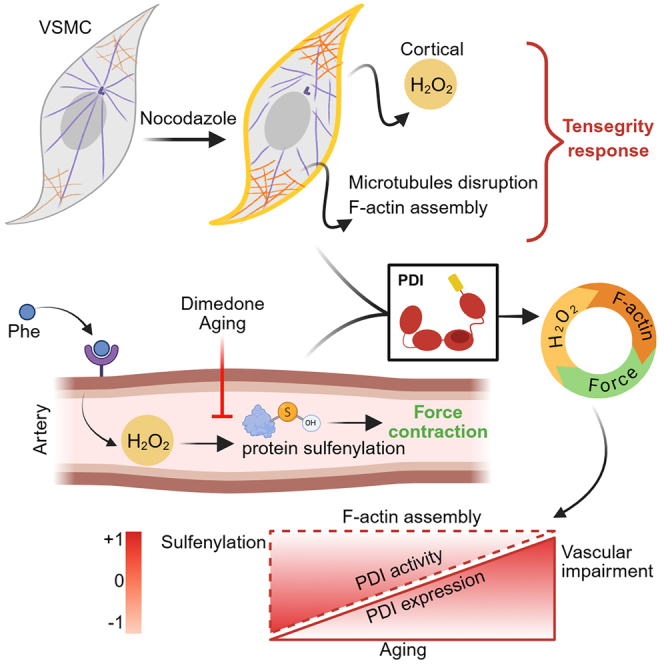

## Introduction

Aging of vascular cells significantly contributes to the overall organismal aging phenotype and is a major independent risk factor for cardiovascular diseases.^1^ While many studies focused on endothelial cells,^2^ aging-related processes also affect the vascular smooth muscle cell. An aged VSMC associated with disturbed arterial stiffness^3^ and, in particular, impaired contractility.^4^^,^^5^^,^^6^^,^^7^ The latter, in particular, leads to disabled adaptive mechanisms that compromise physiological adjustments.^4^ Thus, further studies are necessary to understand the mechanisms underlying aging-associated impairment of vasoconstriction. Cytoskeletal deregulation, mainly of the actin network, lies at the core of such changes.^8^ Importantly, cytoskeleton-linked mechanobiological processes strongly crosstalk with redox-dependent signaling at several levels, from sensing to tissue remodeling.^9^ In particular, an oxidant environment promotes actin polymerization^10^ and enhances contractility.^11^ It is conceivable, thus, that post-translational redox modifications, including e.g., protein sulfenylation, affect actin organization, but the precise role of such an oxidant environment on vascular contractility during aging is unknown. In fact, aging has long been associated with redox derangements, coined as “Free Radical Theory of Aging,”^12^ although such a paradigm has been questioned at several levels.^13^ For instance, redox proteomics analysis depicts that aging is not associated with massive protein oxidation,^14^^,^^15^ indicating a complex interplay between redox processes and aging phenotypes. In this sense, stress-adapting proteins, such as chaperones, may be important for connecting oxidant signaling to specific targets.^16^ Indeed, stress induces loss of chaperone-dependent integrative nodes in protein interaction networks.^17^ We hypothesized that the aging-related impairment of redox and sulfenylation-regulated cytoskeleton dynamics associates with the disruption of chaperone signaling. A particular subgroup of redox chaperones is the protein disulfide isomerases, with prominence of its founding member PDIA1 (or simply PDI). This thioredoxin superfamily protein is mainly located in the endoplasmic reticulum (ER), where it supports oxidative protein folding. Meanwhile, it also exhibits functions out of the ER associated with mechano-regulation, including fine-tuning of cellular force distribution,^18^ integrin regulation^19^ and β-actin organization,^20^ accounting for vascular remodeling modulation.^21^ Of note, the disruption of thiol oxidoreductases, including those from the PDI family, associated with shorter lifespan in yeast.^22^ Also, instability of mechanoregulatory proteins is accelerated by aging,^23^ while PDI protects protein conformation via redox mechanisms during force-induced unfolding under repeated cycles of mechanical stimulation.^24^ PDI regulates mechanisms reported to fine-tune sulfenylation-dependent signaling, including local oxidant generation and redox modulating substrates with the potential to directly act on the actin cytoskeleton. Therefore, we hypothesized that vascular contractile impairment by aging involves the disruption of oxidant/sulfenylation-dependent actin cytoskeleton regulation by PDI.

## Results

In Part 1, we initially validated a model to investigate the redox control of tensegral cytoskeleton responses. Then, in Part 2, we use this model to investigate specific aspects of contractile impairment in aged arteries.

### Part 1

#### Protein sulfenylation regulates vasoconstriction

**Figure 1:**
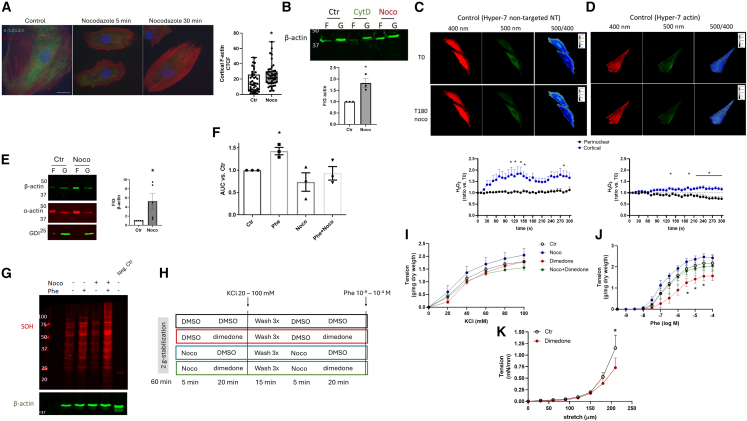
Actin remodeling by nocodazole sustains sulfenic acid dependent vasoconstriction (A) Line cell of rat vascular smooth muscle A7R5 were serum starved for 1 h, treated in serum free conditions with DMSO (Ctr) or nocodazole (Noco 10 μM at 5 or 30 min), and fluorescence of F-actin (red) or α-tubulin (green) was measured. Nuclei were stained with Hoechst 33342 and shown in blue. Scale bars 20 μm. Quantification of cortical actin was measured using ImageJ at 30 min-time point. Graph depicts corrected total cell fluorescence (CTCF). (B) A7R5 cells in control or treated with nocodazole (Noco 10 μM) or cytochalasin D (CytD 5 μM) for 30 min. Filamentous (F) and globular (G) actin pools were separated, and β-actin expression was measured. Graph at the bottom depicts F/G actin vs. the Ctr group. *N* = 3, ∗*p* < 0.05 vs. Ctr (unpaired *t* test). (C–D) A7R5 cells were transfected with H_2_O_2_ sensor Hyper-7 non-targeted (NT, C) or directed to F-actin (D). Twenty-four hours after transfection, cells were serum starved for 1 h. After, cells were treated with nocodazole 10 μM and fluorescence was measured at 400 nm or 500 nm excitation for the detection of reduced or oxidized sensor, respectively. Ratio was calculated using ImageJ (panels at right) comparing perinuclear vs. cortical area. Graphs at the bottom depict 5 min-time course of fluorescence ratio measured at distinct points in the cell perinuclear or cortical regions vs. time zero. ∗*p* < 0.05 Ctr vs. perinuclear (two-way ANOVA followed by Bonferroni post-test). Measurements were performed in 3–6 cells from three independent experiments. (E) Rat aortic rings from control rats were maintained in an organ bath under tension (2 g) for 60 min and treated with Nocodazole 10 μM for 20 min. Tissues were lysed and proceeded for F-G actin separation as described in “A.” Beta actin and α-actin expression were measured. Graph at bottom depicts F/G ratio from β-actin vs. Ctr. RhoGDIα (GDI) depicts the purity of the F or G fractions separation. ∗*p* < 0.05 vs. Ctr (paired *t* test), *n* = 5. (F) H_2_O_2_ measured using Amplex Red in aortic rings in control (Ctr) or treated with phenylephrine (Phe 30 nM), Nocodazole (Noco 10 μM), or both (Phe 30 nM + Noco 10 μM). Incubations were performed during the Amplex Red assay at 10 μM in the presence of horseradish peroxidase (1 U/ml). Relative fluorescent units were corrected by dry weight. Graph depicts the area under the curve from 60 min-measurements. *N* = 3 independent experiments, with 3 rings per experiment. ∗*p* < 0.05 Ctr vs. Phe (one-way ANOVA followed by Dunnett's post-test). (G) Rat aortic rings from control rats were maintained in an organ bath under tension (2 g) for 60 min and treated with dimedone 5 mM, iodoacetamide 5 mM, DTPA 100 μM, and catalase 200 U/ml for 10 min. Immediately after, rings were incubated in lysis buffer supplemented with the same reagents and protease inhibitors cocktail. Rat abdominal aortic rings without incubation with dimedone were used as negative control in each experiment. Expression of protein sulfenylated (SOH) was measured using anti-dimedone and β-actin was used as the loading control. *N* = 2 independent experiments. (H) Aortic rings from rats were maintained in organ bath under tension (2 g) and treated with DMSO (Ctr), nocodazole 10 μM (25 min), phenylephrine 30 nM (20 min) or both (nocodazole was pre-incubated 5 min before phenylephrine stimulation, total treatment 25 min and 20 min, respectively). After treatments, vasoconstriction to KCl and phenylephrine was measured. Three washes with Krebs solution were performed (5 min each) between curves followed by another round of treatments. (I and J) (I) Vasoconstriction to KCl 20–100 mM or (J) phenylephrine (phe 10^−9 - −4^ M). ∗*p* < 0.05 Dimedone vs. Ctr, *n* = 6. Contractions were corrected by arterial dry weight. (K) Myogenic response of third-to fourth-order order mesenteric arteries from normal rats mounted in wire myograph to study the effect of dimedone as described in “H.” ∗*p* < 0.05 Dimedone vs. Ctr, *n* = 4. All graphs in this figure are presented as mean ± SEM.

Microtubule and actin filaments counterbalance themselves as organized, rigid, and contractile mechanical elements, respectively, composing a homeostatic tensegral ensemble.^25^ Thus, we devised a model of microtubule disruption as a stimulus for actin cytoskeleton dynamic remodeling in VSMC in a way independent from external force application. VSMC A7R5 were incubated with the microtubule disrupting agent nocodazole (10 μM–5 or 30 min). This resulted in fast microtubule collapse and promoted increased levels of polymerized (F) vs. soluble (G) β-actin (Figures 1A and 1B quantifications at 30 min).

Using this model, we investigated redox mechanisms involved in actin cytoskeleton remodeling and started by addressing whether F-actin reorganization after nocodazole associates with changes in oxidant levels. First, VSMCs were transfected with the genetically encoded Hyper7 H_2_O_2_ biosensor without any targeting sequence (NT, from non-targeted), which showed that nocodazole leads to increased H_2_O_2_ production at the cell cortex compared with the perinuclear area (Figure 1C). Such a local production of H2O2 is able to impact on actin organization related to integrin signaling^26^ or to the oxidation of actin regulators.^27^ Thereafter, we asked if such increased H_2_O_2_ production occurs in close proximity to actin filaments and used a Hyper-7 sensor directed to F-actin (Hyper-7-actin). Hydrogen peroxide production was also enhanced compared to perinuclear distribution (Figure 1D). The overall response to nocodazole exposure was lower than that of the non-targeted sensor (Figure 1C vs. 1D), which we attribute to the capacity of the F-actin-driving peptide sequence to affect actin dynamics, enhancing the filamentous pool.^28^ Overall, cytoskeleton remodeling by nocodazole promotes increased H_2_O_2_ levels at the cell cortex.

We then turned to investigate aortic rings and showed that F-actin induction by nocodazole was also detected in this tissue (Figure 1E), while the global production of H_2_O_2_ in this scenario was unaffected (Figure 1F). This further suggested that local effects on pro-oxidant signaling rather than global oxidant generation could be involved in F-actin effects. We thus addressed possible roles of protein sulfenylation, which had not yet been investigated concerning the vascular contractile response. First, we addressed protein sulfenylation in response to nocodazole and showed that the global protein sulfenylation pattern was unaffected (Figure 1G). We then investigated the effects of distinct contractile agents. Exposure to phenylephrine, a receptor-dependent contractile agonist, promoted both an increase in H_2_O_2_ generation (Figure 1F) and increased protein sulfenylation (Figure 1G). Meanwhile, such increased production of H_2_O_2_ in response to phenylephrine was not observed if arterial rings were previously exposed to nocodazole (Figure 1F), while co-exposure of phenylephrine and nocodazole accentuated protein sulfenylation (Figure 1G). These results suggest that sulfenylation dependent signaling is important for contractile response and that priming actin assembly drives protein oxidation, bypassing the effects of global generation of H_2_O_2_ during vascular contraction.

Given these results, we hypothesized that a sulfenylation-enriched milieu sensitizes to vasoconstriction. Then, we tested two loss-of-function approaches (protocol described in Figure 1H). The first was dimedone, a carbon-based nucleophilic reagent that covalently binds to sulfenic acid and has been used not only for detection but also for functional purposes.^29^^,^^30^^,^^31^ Interestingly, dimedone disrupted the response to phenylephrine, with reduced sensitivity though negligible effect on maximal response (Figure 1J), while the contraction to potassium chloride (Figure 1I), a receptor independent agonist, was not significantly affected. The inhibitory effect of dimedone was still detected in arteries with endothelium removed (Figure S1), indicating that the role of oxidant signaling mediated by sulfenic acid is likely to account for local intracellular processes in VSMC. However, some contributions of paracrine signaling cannot be excluded as inhibition occurred at a lower concentration range vs. endothelialized aortic rings (Figures 1J vs. S1). These results indicate that sulfenylation positively regulates vascular contraction in large vessels. To further characterize if such an effect also occurs in resistance arteries, the myogenic response was also evaluated in third-to fourth order branches from the superior mesenteric arteries. Incubation with dimedone also decreased tension induced by stretch (Figure 1K), indicating that sulfenylation plays a role in myogenic response as well. We next tested if nocodazole, which upregulates F-actin, affects vascular contraction and the inhibitory effect promoted by sulfenylation neutralization. Nocodazole alone does not affect vascular contraction. However, nocodazole preincubation (10 μM for 5 min, Figure 1J) prevented the inhibitory effect promoted by dimedone in phenylephrine-induced contraction. These findings indicate that priming F-actin assembly bypasses vasoconstriction impairment caused by disrupted sulfenylation.

Collectively, these results demonstrate that actin cytoskeleton remodeling induces local H_2_O_2_ buildup, which supports sulfenylation-dependent signaling and facilitates contractile responses. Meanwhile, the differences in global vs. local H_2_O_2_ generation, when confronted with H_2_O_2_ dependent-signaling by phenylephrine vs. nocodazole, suggest the presence of focalization mechanisms adapting the global redox milieu to downstream protein oxidant targets. One such mechanism may be the effect of PDI, as follows.

#### Association between PSOH and PDI on redox-sensitive F-actin assembly and vascular contraction

**Figure 2:**
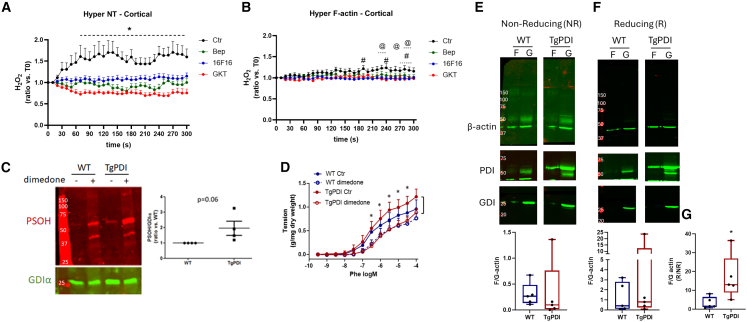
PDI positively regulates the local production of H2O2, vascular sulfenylation, redox-sensitive F-actin assembly, and SOH-sensitive vascular contraction (A and B) (A) A7R5 cells were transfected with H_2_O_2_ sensor Hyper-7 non-targeted (NT, A) or directed to F-actin (B) as described in Figure 1C. Twenty four hours after transfection cells were serum starved for 1 h and treated with DMSO or PDI inhibitors 16F16 3 μM, Bepristat (Bep 15 μM), or Nox inhibitor GKT 40 μM for 30 min. Cells were treated with nocodazole 10 μM, and fluorescence was measured at 400 nm or 500 nm excitation for the detection of reduced or oxidized sensor, respectively. Ratio was calculated using ImageJ/Fiji (panels at right). Graphs depicts 5 min-time course of fluorescence ratio measured at distinct points at cortical region vs. time zero using NT-Hyper (A) or targeted to F-actin (B). ∗*p* < 0.05 Ctr vs. other groups. ^#^*p* < 0.05 16F16 vs. Ctr. ^@^*p* < 0.05 GKT vs. Ctr (both comparisons performed with two-way ANOVA followed by Bonferroni post-test). Measurement was performed in 3–6 cells from three independent experiments. (C) Descending thoracic aorta from wild type (WT) or with PDI overexpression (TgPDI) were lysed in buffer supplemented with reagents described in Figure 1H for the detection of sulfenylated proteins. Graph depicts total lane quantification corrected by RhoGDIα as loading control and normalized vs. WT group. *p* = 0.06 vs. WT vs. TgPDI (paired *t* test). (D) Contraction to phenylephrine of WT or TgPDI mice was measured in control condition or incubated with dimedone 5 mM during 20 min as described previously (Figure 1G). ∗*p* < 0.05 TgPDI Ctr vs. TgPDI dimedone (two-way ANOVA followed by Bonferroni post-test). (E–G) Abdominal aorta or common carotid arteries were incubated in Krebs buffer during 30 min (37°C, CO_2_ 5%) and proceed for F-G separation. Western blot of β-actin was performed in non-reducing (E) or reducing conditions (F). PDI detection was also performed to confirm PDIA1 overexpression. Non-relevant lanes were removed from the original image for clarity. Graphs at bottom depict F/G actin from respective non-reducing (NR) or reducing (R) measurements. (G) Ratio between F/G actin from reducing vs. non-reducing conditions from each experiment. Box and Whiskey graphs were used due to data assumed nonnormal distribution. ∗*p* < 0.05 vs. WT (Mann-Whitney test), *n* = 5. All graphs in this figure are presented as mean ± SEM.

Because PDI is known to support localized oxidant generation connected to vascular remodeling,^21^ we next investigated PDI involvement in local levels of H_2_O_2_. In extended approaches from Figures 1C and 1D, the effect of PDI modulation on local H_2_O_2_ production during cytoskeleton remodeling was tested using distinct PDI inhibitors, namely bepristat-2a and 16F16 (Figure 2A). The induced cortical H_2_O_2_ production during response to nocodazole was prevented by either PDI inhibitor. In addition, the Nox 1–4 inhibitor GKT136901 also prevented nocodazole-stimulated H_2_O_2_ generation (Figure 2A), indicating that the previously described convergence between PDI and NADPH oxidase^32^ is a likely mechanism of oxidant generation during cytoskeleton remodeling. Analogous effects were also shown upon targeting Hyper to F-actin (Figure 2B). The combination of Nox and PDI neutralization does not enhance the inhibitory effect (Figures S2A and S2B), suggesting that both act within the same oxidant-generating system, in this case supporting NADPH oxidase complex activation during the response to nocodazole.

Previous studies showed that PDI overexpression increases oxidant production in VSMC under angiotensin II or PDGF treatment and in arteries from mice overexpressing PDI (TgPDI).^32^^,^^33^^,^^34^ Thus, we investigated if protein sulfenylation is affected in the aorta of TgPDI mice. Global vascular PSOH is increased by genetic PDI overexpression compared with WT mice (Figure 2C). In addition, TgPDI aortas exhibited a significant contribution of sulfenyl residues to phenylephrine-induced contractility, with an accentuated inhibition of contraction by dimedone vs. WT mice aortas (Figure 2D). This further reinforces a role for protein sulfenylation during receptor dependent contraction. Because β-actin reportedly interacts with PDI in high molecular weight complexes detected only in non-reducing conditions,^20^ we conducted analysis of F/G-actin in distinct redox conditions to study potential differences in actin detection at its canonical molecular weight (42 kDa). In TgPDI, we observed significantly higher levels of F actin (i.e., F/G ratio) in reducing conditions, when normalized for corresponding non-reducing conditions (Figure 2G). This is consistent with higher recovery of oxidized β-actin to its canonical molecular weight, although TgPDI does not depict absolute differences in F/G actin in non-reducing (Figure 2E) or reducing conditions (Figure 2F). Acute PDI induction in aortic rings (Figure S3A), increases contraction to phenylephrine (Figure S3C) but not to KCl (Figure S3B) in association with increased actin polymerization (Figure S3D). Together, these results suggest that PDI acts as a regulator of redox-dependent F-actin remodeling in vascular tissue, supporting actin fiber buildup.

#### Mechanisms involved in the redox regulation of the actin cytoskeleton by PDI

**Figure 3:**
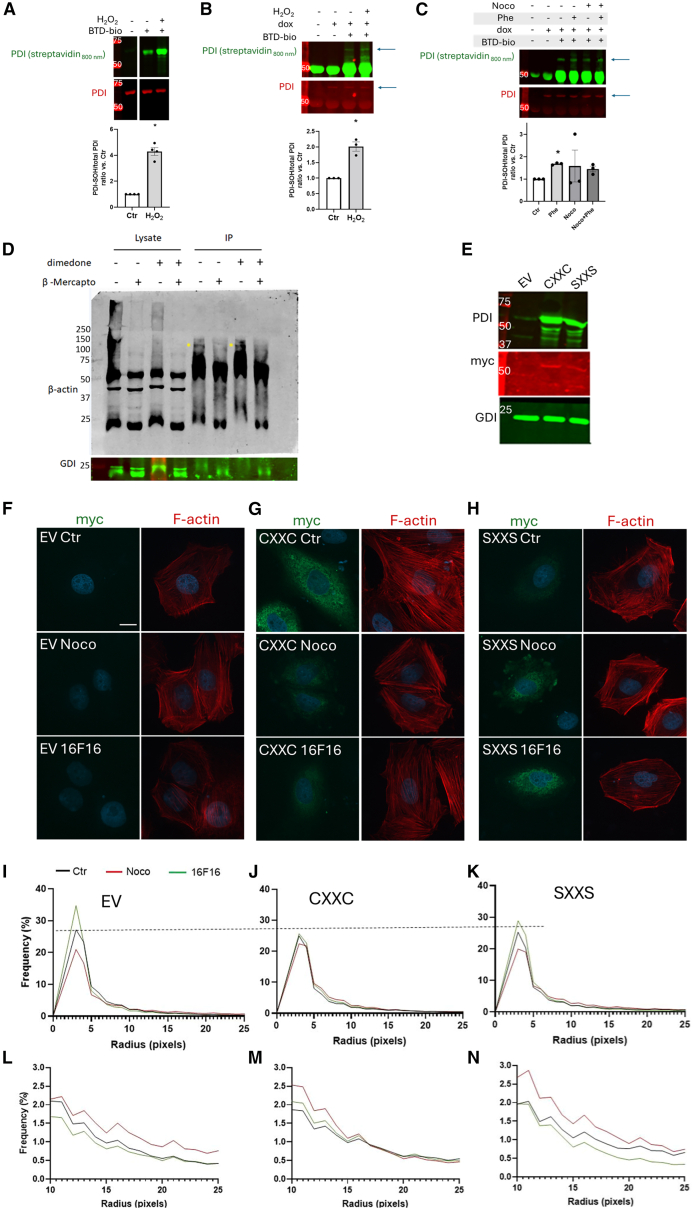
Potential mechanisms supported by PDI on β-actin redox regulation during cytoskeleton assembly (A) Pre-reduced recombinant rat-PDI 5 μM was incubated or not with BTD-bio 100 μM in the absence or presence of H_2_O_2_ 100 μM during 1 h. PDI sulfenylation was detected with streptavidin conjugated with a fluorophore (streptavidin 800 nm) and total PDI with anti-PDI. ∗*p* < 0.05 vs. Ctr, *n* = 4 (paired *t* test). (B) VSMC with the conditional expression of PDI (myc-tag) were treated or not with doxycycline (dox) for 24 h. After serum starvation 1 h, cells were maintained in control condition or exposed to H_2_O_2_ μM during 30 and lysed in buffer supplemented (or not) with BTD-bio (see “STAR Methods” for more details). PDI containing myc-tag was immunoprecipitated (anti-myc) and SOH fraction was detected using Streptavidin conjugated with fluorophore. The detection of PDI was confirmed with a specific PDI antibody. Arrows indicate PDI. ∗*p* < 0.05 vs. Ctr, *n* = 3 (paired *t* test). (C) As described in “B,” VSMC with the conditional expression of PDI were stimulated with nocodazole, phenylephrine, or both to measure PDI sulfenylation. ∗*p* < 0.05 vs. Ctr, *n* = 3 (one-way ANOVA followed by Dunnett's post-test). (D) Aorta abdominal from WT C57Bl/6 mice lysed in RIPA buffer supplemented or not with dimedone 5 mM. One hundred micrograms of proteins were immunoprecipitated with anti-PDI rabbit and blotted against β-actin mouse in reducing (+β-mercaptoethanol) or non-reducing (-β-mercaptoethanol) conditions. RhoGDIα was used as a loading control. Representative image from 2 independent experiments. (E) A7R5 cells were transfected with empty vector (EV) or with plasmid coding WT rat PDI (CXXC) or mutant PDI with the two catalytic cysteines at a and a'-domains mutated to serine (SXXS). Both exogenous PDIs contain a C-terminal myc tag. Representative Western Blot depicting PDI, myc-tag or RhoGDIα performed 24 h after transfection. (F–H) Immunofluorescence of VSMC as described in “E,” treated with nocodazole 10 μM, 16F16 3 μM or DMSO as control. Left panels depict myc (green) and right F-actin (red). Nuclei are shown in blue. Diameter of actin fibers was measured using DiameterJ from ImageJ/Fiji. (I–N) Plots at the bottom show the frequency distribution of fiber thickness with the whole thickness range (0–25 pixels, I–K) or focused on thicker fibers (10–25 pixels, L–N). Reference dotted line points to the peak of frequency of EV cells in the control condition. Measurements were performed in 40 cells from three independent experiments. Graphs depict a mean histogram. All other graphs in this figure show mean ± SEM.

Our results so far suggest that PDI-dependent global sulfenylation may be upregulated during vascular contraction, but whether PDI itself is sulfenylated in this condition is unclear. To address this issue, we first validated a modified version of the sulfenylation sensor Benzo[c][1,2]thiazine (BTD) tagged with biotin (BTD-bio, Figure S4). Sulfenylated PDI was detected using recombinant protein and in VSMC with PDI overexpression (PDI-myc) under H_2_O_2_ exposure (Figures 3A and 3B), while this signal was decreased by dimedone (Figure S5). Then, we showed that PDI sulfenylation was induced in VSMC stimulated with phenylephrine but not in nocodazole treated cells (Figure 3C), indicating that vasoconstrictor agonists induce PDI sulfenylation.

Redox regulation of β-actin by PDI was shown to support cytoskeleton assembly and cell adhesion in the megakaryoblastic leukemia cell line (MEG-01).^20^ To study if such interaction also occurs in vascular cells and involves protein sulfenylation, PDI/β-actin immunoprecipitation studies were conducted in aortic tissue in distinct redox conditions. First, pre-incubation with dimedone prevented PDI/β-actin interaction (Figure 3D). Second, we showed that β-actin was undetectable at its canonical molecular weight (42 kDa) in non-reducing conditions, suggesting a possible disulfide-dependent complex between PDI/β-actin (yellow arrows). Thus, the interaction between PDI and β-actin in aortic tissue involves thiol sulfenylation and secondary reactions. While PDI helps sustaining local H_2_O_2_ production and global sulfenylation during actin remodeling and contractile stimulation, respectively, in specific contexts, it may also limit actin sulfenylation and induction of high molecular weight (HMW) complexes containing sulfenylated actin by H_2_O_2_ exposure (Figure S6). We next asked whether the effects of PDI on actin remodeling are dependent on its catalytic thiols. For that, we conducted experiments with wild type PDI (CXXC) and mutant PDI without the active cysteines (SXXS) in both a-domains. Transfection of either CXXC or SXXS PDI cDNA in VSMC expectedly increased PDI levels (Figure 3E), as detected by antibodies targeted to PDI or myc-tag. Then, we analyzed the pattern of fiber thickness organization, a proxy for F-actin organization, in the control condition or after incubation with nocodazole or the PDI inhibitor 16F16. The images in Figures 3F–3H and corresponding graphs representing the global fiber profile show that nocodazole diminished the extent of actin fiber thinning in EV (Figure 3I), with increased frequency of thicker fibers (Figure 3L). In contrast, PDI inhibition accentuated fiber thinning (Figure 3I), with a lower frequency of thicker fibers (Figure 3L). Overexpression of WT(CXXC) (Figures 3G–3J and 3M), but not SXXS PDI (Figures 3H–3K and 3N), prevented the effects of 16F16. PDI SXXS transfection led to the highest increase in thicker fibers compared to control and nocodazole conditions (Figure 3N). Thus, it is likely that both thiol oxidoreductase and chaperone effects (per se independent of catalytic thiols) may support either F-actin assembly, composing the complex actin processing^35^ or signaling upstream to F-actin assembly.^36^

### Part 2

#### PDI overexpression restores aging-associated contractile dysfunction

##### Aging-associated contractile dysfunction is reversed in PDI-overexpressing mice

**Figure 4:**
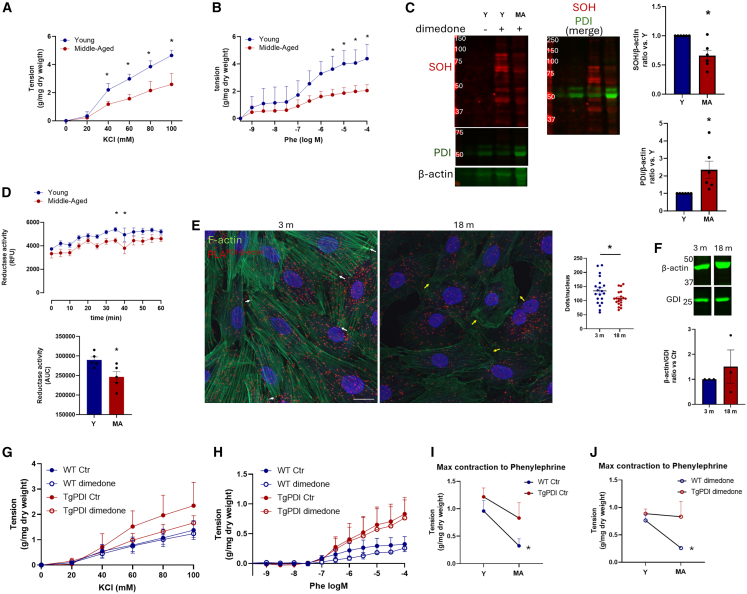
PDI overexpression restores aging-associated contractile dysfunction (A and B) (A) Contraction to KCl 20–100 mM or phenylephrine (Phe, 10^−9 - −4^ M, B) of thoracic aortic rings from C57Bl/6 mice of 3- or 12-month age. ∗*p* < 0.05 vs. 3 vs. 12 m (two-way ANOVA followed by Bonferroni post-test), *n* = 5. (C) Western blot detection for sulfenylated (SOH), PDIA1 or β-actin. Graphs at right depicting global SOH (top) and PDI (bottom). Values were normalized by β-actin and expressed as a ratio vs. 3 m ∗*p* < 0.05 vs. 12 m (paired *t* test), *n* = 5. (D) Fluorescent di-eosin-GSSG reductase activity was measured in aortic homogenates from young (3 m) or middle-aged (14 m) mice. (Top) Graph depicting kinetic measurement during 60 min and (bottom) area under the curve from each experiment, *N* = 5, ∗*p* < 0.05 Young vs. Middle-aged (paired *t* test). (E) Proximity ligation assay (PLA) was performed in VSMC extracted from rat aorta at 3 m or 18 m age to detect proximity between PDI and β-actin. Red dots represent PLA positive signal, F-actin in green, and nucleus in blue. Scale bars 20 μm. Graph at right depicts the number of PLA positive counts per nucleus of 20 measurements from two independent experiments. ∗*p* < 0.05 3 m vs. 18 m (paired *t* test). (F) β-actin expression in VSMC from rat aorta at 3 m or 18 m age. Non-relevant lanes were removed from the original image for clarity. Graph depicts β-actin normalized by GDI and expressed as a ratio vs. 3 m (G–J) (G) Contraction to KCl 20–100 mM (H) or phenylephrine (Phe, 10^−9 - −4^ M, B) of thoracic aortic rings from WT or TgPDI mice with 12-month age in control (Ctr) or incubated with dimedone 5 mM 20 min. Data from Figure 2D were used to compare the maximum contractile response to phenylephine of WT or TgPDI mice at 3 m vs. 12 m old from the control condition (I) or with dimedone incubation (J). ∗*p* < 0,05 WT 3m vs. WT 12 m (unpaired *t* test). N = 3–4. All graphs in this figure are presented as mean ± SEM.

In Part 2 of our results, we address the hypothesis that aging-induced vascular dysfunction associates with disruption in mechanisms related to PDI-dependent sulfenylation. We first confirm that aging impairs vascular contraction by comparing young (Y, 3 months) vs. middle-aged (MA, 12–14 months) Figure 4A and 4B) mice. This is in agreement with studies showing lower contractility and myogenic response during aging.^4^^,^^37^ Importantly, contractile dysfunction was associated with the lower detection of overall protein sulfenylation in aorta of MA mice (Figure 4C). Somewhat unexpectedly, PDI expression was increased by aging (Figure 4C). To the best of our knowledge, increased PDI expression during aging processes has never been reported in blood vessels, although it has been previously shown in mouse liver and in aged human mesenchymal stem cells.^38^ We reasoned then that PDI function rather than expression could be impaired. Indeed, PDI function was shown previously to be disrupted in liver homogenates from old rats.^39^ Thus, we investigated the reductase activity of PDI (and additional oxido-reductases) by measuring the fluorescence increases induced by di-eosin-GSSG reduction. Importantly, aortic homogenates from MA mice presented lower activity (Figure 4D), thus suggesting that PDI inactivation, despite increased expression, may be involved in aging-induced contractile dysfunction. Analyses at additional ages (9 and 24 months) are presented at Figure S7, indicating that contractile dysfunction was more prominent at the MA stage (12 months).

To further understand whether aging-related hypocontractility involves PDI effects on cytoskeleton organization, we investigated, in VSMC from young (3 m) vs. middle-aged rats (18 m), the interaction of PDI with β-actin using proximity ligation (PLA) assays. Interestingly, PLA positive dots, which represent points of close proximity (<40 nm) between the interacting partners PDI and β-actin, decreased in VSMC from MA mice (Figure 4E). Also, aging is associated with decreased F-actin staining (Figure 4E), which is in accordance with aging-promoted loss of contractile phenotype.^40^ In addition, conglomerates with the misalignment of F-actin from MA mice cells presented weak PLA signals (yellow arrows) as opposed to the enriched signal at the well-organized filaments at the actin fiber tips (white arrows). Of note, the amount of β-actin expression does not change with aging (Figure 4F).

We then interrogated whether the deregulation of such interplay between PDI, vascular SOH levels, and cytoskeleton interaction underlies aging-related vascular dysfunction. For that, we studied, in arteries from 12-month-old WT or TgPDI mice, the effects of dimedone on contractile responses to KCl (Figure 4G) and phenylephrine (Figure 4H). Our results indicated that genetic PDI overexpression prevented aging-associated contractile dysfunction, evident by less impairment of maximal contraction to phenylephrine in 12 vs. 3-month-old mice (Figure 4I). Dimedone incubation, which did not show robust contractility impairment in young WT mice, promoted age-associated hypocontractility in aged WT mice. Meanwhile, dimedone-incubated arteries from young TgPDI mice showed accentuated loss of contraction vs. untreated vessels. Meanwhile, the contractility of dimedone-exposed arteries from aged TgPDI mice was essentially preserved vs. corresponding young mice, indicating that genetically upregulated PDI associates with loss of a sulfenylation-dependent component of aging-related hypocontractility (Figure 4J). Thus, chronic PDI overexpression prevents the additive effect of aging on contractile impairment promoted by blocking sulfenylated proteins.

##### Genetic PDI overexpression prevents alterations in vascular viscoelastic properties during aging

**Figure 5:**
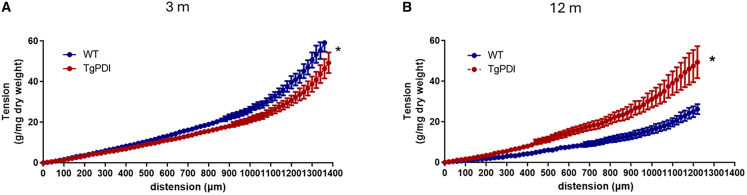
PDI overexpression prevents aortic viscoelastic alterations promoted by aging (A and B) Aortic rings from WT or TgPDI mice at 3 months old (A) or 12 months old (B) were maintained in physiological solution without calcium, supplemented with EDTA 3 mM and sodium nitroprusside 100 μM. Vessels were incrementally stretched with a 50 μm-step. Graphs depict the stretch tension curve. ∗*p* < 0.05 WT vs. TgPDI (two-way ANOVA followed by Bonferroni post-test), *n* = 4.

Having shown that PDI overexpression prevents aging-related vascular hypocontractility, we next investigated whether corresponding aging-related alterations also extend to vascular viscoelastic properties, since PDI reportedly preserves viscoelastic properties in models of aortic disease^41^ and injury repair.^21^ For that, we analyzed stretch-tension curves under incubation with calcium-free Krebs buffer supplemented with SNP 100 μM and cytochalasin D 5 μM (used to inhibit vasomotor tone and actin depolymerization, respectively). In young 3-month-old mice, arteries from TgPDI exhibited lowered tension during stretch vs. control (i.e., lower rigidity; Figure 5A). Conversely, at 12 months of age, such an effect was inverted, with arteries from TgPDI exhibiting viscoelasticity similar to that of young mice, while arteries from controls evolved toward an impaired rigidity, lower than that of TgPDI arteries (Figure 5B). This is consistent with the possibility that chronically, PDI overexpression preserves vascular structure, potentially indicating that sustaining the contractile responses at a younger age may translate into a long-term balanced vascular remodeling.

## Discussion

Our results suggest that sulfenylation-dependent mechanisms support vascular contraction, acting in connection with actin redox organization orchestrated by PDI regulation. However, rewiring of this integrative mechanism contributes to aging-related vascular dysfunction and structural remodeling.

Mechanobiological processes closely interplay with induced oxidant generation and adaptive redox signaling via the oxidation of targets such as integrins^18^ and actin regulators.^27^^,^^42^ Our data adds additional evidence indicating that aging-associated cumulative lifelong alterations in these processes may affect homeostatic set point adjustments both for active and passive elements. These results further reinforce that aging promotes targeted changes in specific redox signaling outcomes, which, to some extent contrasts with the idea of a widespread global oxidation proposed by the “Free Radical Theory of Aging.”^12^ In fact, recent data indicate organ-specific rewiring of the redox proteome during aging.^15^ Indeed, aging-associated hypocontractility was associated with decreased, rather than increased protein sulfenylation, resulting in impaired force production. In this scenario, our data indicate that local, rather than global, H_2_O_2_ levels account for disrupted cystoskeleton rearrangements following a tensegral collapse induced by microtubule depolymerization. This suggests that external mechanical forces may not be necessary to promote redox-dependent mechanoadaptative remodeling. In fact, global interventions targeting sulfenylation^43^ were unsuccessful in delaying aging effects.^44^ Our study focused on middle-aged rodents, which in rats is representative of 12–18 months, in mice 10–14 months,^45^ and in humans 38–47 years,^46^ the latter documented to coincide with a first wave of accelerated aging.^47^^,^^48^ Investigating alterations at this point may contribute to knowledge of early phases of function decay,^48^ opening opportunities for more precocious detection and interventions.

An important finding of our study is to show that preserving PDI function is important to keep vascular contraction during aging. At first sight, this could be attributed to general PDI effects as a redox chaperone related to increased resilience to derangements of proteostasis. While true, our data suggest a more focused mechanism related to actin cystoskeleton regulation, more specifically, a likely direct regulation of β-actin through sulfenylation dynamics. Although sulfenylation-dependent mechanisms drive ancestral signaling controlling life span, for example, switching Ire1α from ER stress activation to stress resistance,^49^ functional endpoint impairments promoted by disrupted sulfenylation control during aging are lacking. The differential sensitiveness of dimedone on contraction impairment by PDI overexpression at distinct ages reinforces the role of regulatory ER-related aging mechanisms involving protein sulfenylation. The mechanisms for PDI interplay with sulfenylated proteins and how this affects the cytoskeleton can involve several pathways. PDI was shown by us to support growth factor-dependent oxidant Nox signaling, acutely accounting for increased VSMC migration and structural remodeling.^21^ Extracellular PDI closely modulates fine-tuning adjustments of force distribution.^18^ However, sustained PDI overexpression is associated with a switch toward VSMC redifferentiation to a contractile phenotype.^34^ In fact, TgPDI arteries exhibit markers of VSMC over-differentiation, indicating a twisted set-point. In parallel, we reported that PDI prevents aging promoted viscoelastic impairment, which likely involves extracellular matrix reorganization. Indeed, PDI is closely involved in extracellular matrix protein processing and organization during vascular remodeling.^21^ Overall, such a differentiated phenotype and ensuing viscoelastic rewiring could help preserve the aging-related contractile behavior. However, this requires further studies. Additional mechanisms mediating PDI effects may also relate to calcium dynamics within the ER.^50^

Increased γ-actin and decreased α-actin content reportedly impair traction force generation in middle aged VSMCs.^40^ Here we report that the non-muscle cytosolic β-actin is also relevant for vascular aging. Interestingly, β-actin sulfenylation is an intermediate step for S-glutathionylation,^51^ known to decrease actin polymerization.^52^ In addition, in VSMC, β-actin sulfenylation seems restricted to the F-actin pool, supporting its interaction with functional partners such as vinculin during cell adhesion.^26^ Mechanisms supporting F-actin SOH may involve its proximity with oxidant sources such as NADPH oxidase Nox4 in VSMC, in line with our results showing attenuated cortical production of H_2_O_2_ with Nox inhibitor. Importantly, PDI reportedly interacts with β-actin Cys374 in megakaryocytes, forming a redox complex at around 110 kDa.^20^ Here, a similar redox complex containing PDI was shown to be increased during aging (Figure S8). In addition, our results suggest that PDI-β-actin interaction is dependent on sulfenylation in vascular tissue, and its interaction is impaired in middle-aged VSMC. However, PDI-SOH reportedly increases in high molecular weight redox complexes in the lungs of mice exposed to cigarette smoke.^53^ Thus, further studies are required to elucidate if such complex formation relates to PDI inactivation or represents transitory complexes with substrates, such as β-actin, to sustain actin filament assembly.

Our results further confirmed previous reports indicating that PDI can be sulfenylated, although this was only detectable after PDI overexpression in connection with hydrogen peroxide or contractile stimulation. Indeed, PDI sulfenylation can occur either by directly reacting with 2-electron oxidants, such as H_2_O_2_, or by the desulfenylation of interacting substrates, as proposed previously for Drp1.^54^ Here, the desulfenylase role of PDI was reinforced by limiting actin-SOH levels. Moreover, there are additional candidates targeted by sulfenylation, potentially regulated by PDI, with further implications for the subjects addressed in the present study (Figure S9). However, the low rate constant of PDI with H_2_O_2_ (17.3 M^−1^ s^−1^)^55^ as compared, for example, with peroxiredoxins and glutathione peroxidase (c.a. 10^7^ M^−1^ s^−1^), or the lower reducing power compared with thioredoxin,^56^ raises questions as to whether both mechanisms can occur broadly on a sustained basis. However, the potential PDI localization in oxidant-enriched environments (e.g., close to an enzymatic source) or close to a sulfenylated substrate would compensate such a kinetic disadvantage. In fact, our previous study highlighted the presence of PDI within vesicles surrounded by F-actin in VSMC,^18^ so that the close proximity and high concentration promoted by such an enclosed environment would support PDI/β-actin interaction. Our results indicate that the interaction is disrupted by blocking SOH and is not resolved in non-reducing conditions, reinforcing a mechanism based on SOH for their interaction. Furthermore, our recent results suggest that PDI may translocate to the cytosol to some extent, apparently as a process regulated by the ER transmembrane chaperone DNAJB12.^31^

**Figure 6:**
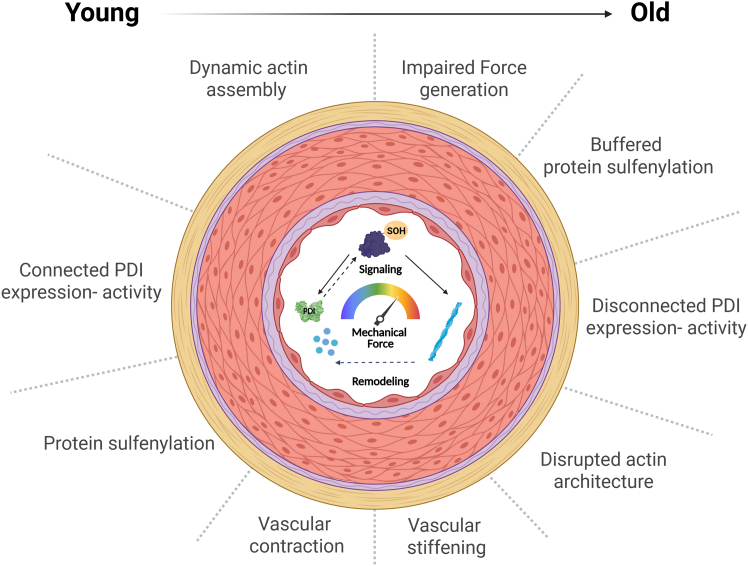
PDI supports actin remodeling through redox processes involving thiol sulfenylation-dependent mechanisms In healthy conditions (young), such dynamic control optimizes local oxidant generation to promote actin cytoskeleton assembly to generate force contraction, while aging processes present disconnected PDI expression with activity, downregulating SOH signaling, disrupting F-actin organization and impacting force generation.

In summary, our data indicate that aging-related vascular hypocontractility emerges in a context of disrupted redox mechanisms involving the effects of a sulfenylation-enriched milieu on the interaction between actin and PDI. On an extended perspective, since chaperones in general are important to orchestrate network signaling,^17^ it is possible that PDI activity acts to control homeodynamic processes coupling protein (de)sulfenylation with (dis)assembly of actin filaments, a process that becomes dysfunctional under long-term exposure of vessels to such oxidant challenge (Figure 6).

### Limitations of the study

Approaches used in the present study to evaluate mechanisms supported by PDI involved mostly gain-of-function tools, such as PDI-overexpressing mouse models, plasmidial transfection in cells and tissues, and cells with conditional PDI overinduction. However, loss-of-function studies were limited to pharmacological inhibitors, which are feasible for approaching more acute events such as H_2_O_2_ generation or actin dynamics. Although these models showed a clear contribution of the VSMC PDI to vascular contraction, they do not allow a clear appreciation of the potential roles of endothelial cell PDI, via pathways affecting NO or endothelin mechanisms. Thus, further studies using cell-specific, preferentially inducible models of PDI deletion would be needed to clarify the roles of each cell type in those effects.

## Resource availability

### Lead contact

Requests for further information and resources should be directed to and will be fulfilled by the lead contact, Leonardo Yuji Tanaka (leonardotanaka@yahoo.com.br).

### Materials availability

All unique/stable reagents generated in this study are available from the lead contact following a completed materials transfer agreement.

### Data and code availability

• Data: All data reported in this article are available from the lead contact upon request.
• Code: No original code was generated in this study.
• Other items: Any further information needed to reanalyze the data presented in this study can be obtained from the lead contact upon request.

## Acknowledgments

We are grateful to Dr. Mario Costa Cruz (Confocal Microscopy Facility, CEFAP-USP) for his technical assistance. This work was supported by: Young Investigator-10.13039/501100001807FAPESP (Fundação de Amparo à Pesquisa do Estado de São Paulo) grant 2018/07230-5 to LYT; CEPID (Centro de Pesquisa Inovação e Difusão)/FAPESP grant 2013/07937–8 to F.R.M.L.; 10.13039/501100001807FAPESP grant 2023/07341-0 to R.S.G.; 10.13039/501100001807FAPESP grant 2023/03079-9 and 10.13039/501100003593CNPq (National Council for Scientific and Technological Development) grant 407911/2021-9 to A.A.M.; 10.13039/501100001807FAPESP scholarship grant to C.M.P. 2019/18448-4, G.S.G. 2019/18448-4, LTY 2020/04280-1, 2018/07511-4 to T.C.B., 2020/03838-9 to L.T., 2021/14131-6 to J.M.F.S., 2023/14702-9 to P.C.B.

## Author contributions

Conceptualization, L.Y.T.; formal analysis and investigation, L.Y.T., L.F.G., R.C.M., G.S.G., C.M.P., V.D., J.M.F.S., T.C.B., L.T., P.V.S.O., B.P.S., S.K.T., P.C.B., A.A.M., R.S.G., D.K., and A.B.; curation, L.Y.T. and I.C.V.; funding acquisition, L.Y.T. and F.R.M.L.; methodology, L.Y.T., L.T., P.V.S.O., B.P.S., I.C.V., A.B., and K.C.; project administration, L.Y.T.; resources, L.Y.T., K.C., and F.R.M.L.; software, L.Y.T., R.S.G., and I.C.V.; supervision, L.Y.T., S.K.T., A.A.M., K.C., and F.R.M.L.; validation, L.Y.T. and K.C.; visualization, L.Y.T.; writing – original draft, L.Y.T.; writing – review and editing, L.Y.T. and F.R.M.L.

## Declaration of interests

The authors declare no competing interests.

## STAR★Methods

### Key resources table

**Table:** 

REAGENT or RESOURCE	SOURCE	IDENTIFIER
**Antibodies**		

Anti-actin	Cytoskeleton	Cat #AAN01; RRID:AB_10708070
Anti-smooth muscle α-actin	Sigma	Cat #A2547; RRID:AB_476701
Anti-β-actin	Sigma	Cat #A5441; RRID:AB_476744
Anti-Cysteine Sulfenic Acid	Millipore	Cat #072139; RRID:AB_1977145
Anti-mouse-800 nm	Li-Cor	Cat #926-32212; RRID:AB_621847
Anti-PDI mouse clone RL90	Invitrogen	Cat #MA3-019; RRID:AB_2163120
Anti-PDI rabbit Enzo	Enzo	Cat#SPA-890; RRID:AB_2313814
Anti-rabbit 700 nm	Li-Cor	Cat#926–68073; RRID:AB_10954442
Anti-RhoGDI Abcam	Abcam	Cat#ab135252; RRID:AB_2893181

**Bacterial and virus strains**		

*E.coli* strain *BL21(DE3) –* Novagen	Sigma	Cat #69450-3
pET28a - Novagen	Sigma	Cat #69864-3

**Chemicals, peptides, and recombinant proteins**		

16F16	Sigma	Cat #SML0021
Actin	Cytoskeleton	Cat #AKL99
Amplex red	Invitrogen	Cat #A12222
Aprotinin	Sigma	Cat #A1153
Bepristat	Sigma	Cat #SML1925
BSA	Sigma	Cat #A7906
BTD-bio	gift from Dr. Kate Carroll	This paper
Catalase	Boehringer Mannheim	Cat #106836
Cytochalasin D	Sigma	Cat #C8273
Dimedone	Sigma	Cat #D153303
DMEM high glucose Medium	Gibco	Cat #121000046
DMEM low glucose Medium	Gibco	Cat #31600034
Doxycycline	Sigma	Cat #D9891
Diethylenetriaminepentaacetic acid (DTPA)	Sigma	Cat #D6518
Ethylenediaminetetraacetic acid (EDTA)	Sigma	Cat #E4884
Eosin-5-isothiocyanate	Molecular Probes	Cat #E−18
Fetal bovine serum	Gibco	Cat #12657029
GKT136901	Merck	Cat #534032
H_2_O_2_	Merck	Cat #107210
Hoestch33342	Sigma	Cat #B2261
Iodoacetamide	GE Healthcare	Cat #RPN6302
Isopropyl ß-*d*-1-thiogalactopyranoside (IPTG)	Sigma	Cat #I5502
Kanamycin	Sigma	Cat #K4000
Leupeptin	Sigma	Cat #L2023
Lysozyme	Sigma	Cat #L6876
Nocodazole	Sigma	Cat #M1404
Nonfat milk	Bio-Rad	Cat #1706404
Phalloidin 488 nm	Invitrogen	Cat #A12379
Phenylephrine	Sigma	Cat #P6126
Phenylmethylsulfonyl fluoride	Sigma	Cat #P7626
Potassium Chloride	Sigma	Cat #P4504
Streptavidin IR dye 800 nm	Li-Cor	Cat #926–32230
β-mercaptoethanol	Sigma	Cat #M7154

**Critical commercial assays**		

Duolink kit	Sigma	Cat #DUO92101
F/G actin separation kit	Cytoskeleton	Cat #BK037

**Experimental models: Cell lines**		

Conditional PDI overexpressing Rabbit Aortic Smooth Muscle Cell	Fernandes et al.^34^	N/A
A7r5	BCRJ	Cat #0034

**Experimental models: Organisms/strains**		

Mouse: Strain FVB WT	FMUSP animal facility	BIOT3882
Mouse: Strain FVB TgPDI	FMUSP animal facility	BIOT3882
Mouse: Strain C57BL/6	FMUSP animal facility	SPF3004
Rat: Strain Wistar	InCor animal facility	N/A

**Recombinant DNA**		

pET28a hPDI - Novagen	Sigma Aldrich	Cat #69864-3
Empty Vector – pcDNA 3	Gift from Dr. Xavier Neto^33^	N/A
Rat PDI CXXC cDNA - pcDNA 3	Gift from Dr. Tomohiro Nakamura^57^	N/A
Rat PDI SXXS – pcDNA 3	Gift from Dr. Tomohiro Nakamura^57^	N/A
Hyper7-actin	gift from Dr. Vsevolod Belousov^58^	N/A
Hyper7 NT	gift from Dr. Vsevolod Belousov^58^	N/A

**Software and algorithms**		

ImageJ/Fiji	Schindelin et al.^59^	https://imagej.net/software/fiji/; RRID:SCR_002285
ZEN lite	ZEISS	https://www.zeiss.com/microscopy/en/products/software/zeiss-zen.html; RRID:SCR_023747
LabChart 8	Danish Myo Technology	ADInstruments
GraphPad Prism 9	GraphPad	RRID:SCR_002798

### Experimental model and study participant details

#### Ethics approval and experimental models

This study was approved by the Ethics Committee of the University of São Paulo Medical School (CEUA 1219/2019), and all experiments were conducted in accordance with relevant regulatory standards and institutional guidelines. Control male Wistar rats were used between 3 and 6 months old. Male mice strains C57BL/6 or FVB either Wild Type (WT) or with PDI overexpression (TgPDI) were used from 3 to 24 months old. TgPDI model was achieved by lentiviral transduction of rat PDIA1 gene in FVB embryos as previously described.^34^ Comparative studies of TgPDI vs. WT mice were performed using mice from the same litter. Both species were housed in cages containing 3–5 animals, under controlled temperature (22 ± 2 °C), humidity (40–60%), and a 12:12 h light/dark cycle, with free access to standard laboratory chow and water *ad libitum*.

Tissue collection from both rats and mice were performed after deep anesthesia with isoflurane followed by euthanasia with CO_2_.

### Method details

#### Immunoblotting

Conventional western blots were performed using lysis buffer supplemented with protease inhibitors (aprotinin 1 μg/mL, leupeptin 1 μg/mL and PMSF 1 mM). For detection of sulfenylated proteins, in addition to protease inhibitors mentioned, lysis buffer was supplemented with iodoacetamide 10 mM, DTPA 100 μM, catalase 200 U/ml and dimedone 5 mM. Negative controls were performed without dimedone in all assays. Ten to 30 μg of proteins from total lysate were separated in reducing or non-reducing conditions (with or without β-mercaptoethanol 5% (710 mM) in sample buffer) through electrophoresis in polyacrylamide gel (usually 12%). Proteins were transferred in wet conditions to nitrocellulose, blocked with non-fat milk 5%, incubated overnight with primary antibodies followed by secondary fluorescent antibodies.

#### Protein expression and purification

Human PDI cloned in pET28a vector were transformed into *E. coli* strain BL21(DE3) and growth in LB medium supplemented with 50 μg/mL kanamycin at 37°C. When the O.D. 600 nm reached 0.6–0.8 the pET28a hPDI, protein expression was induced adding 0.1 mM IPTG for 6h. Cells were harvested by centrifugation at 3,000 rpm for 20 min at 4°C. The pellet was resuspended in resin equilibration/washing buffer (Na_3_PO_4_ 50 mM and NaCl 300 mM, pH 7.0) with lysozyme (200 μg/mL) and PMSF (phenylmethylsulfonyl fluoride, 2 mM) followed by sonication at 70% amplitude during 15 min (5 s ON pulse/5 s OFF pulse). The lysate was centrifugated at 15,000 rpm for 45 min at 4°C. Immobilized metal affinity resin (TALON affinity resin) was used to purify the lysate. Proteins were eluted with increased concentrations of imidazole. Followed by the dialysis using an equilibration/washing buffer (Na_3_PO_4_ 50 mM and NaCl 150 mM, pH 7.0) to remove imidazole. SDS-PAGE were used to confirm the elution and the protein expression. The protein was concentrated by ultrafiltration (Amicon Ultra, cut-off 50 kDa, Millipore).

#### Detection of PDI and actin sulfenylation

Line cell of vascular smooth muscle cells from rabbit aorta containing inducible doxycycline (dox) activated sequence to overexpress PDI (containing myc-tag) were seed at 2x10^6^ cell/100 mm^2^-plate. After 24 h of dox 1.5 mg/mL treatment, cells were serum starved (1 h) and lysed in radioimmunoprecipitation assay (RIPA) buffer containing Tris-HCl 10 mM, EDTA 1 mM, EGTA 0.5 mM, Triton X-100 1%, Sodium Deoxycholate 0.1%, SDS 0.1%, NaCl 140 mM, pH 8.0, supplemented with protease inhibitors (described above), iodoacetamide 10 mM, DTPA 100 μM, catalase 200 U/mL and benzothiadiazole conjugated with biotin (BTD-bio, gift from prof. Kate Carroll, 500 μM). Negative controls without BTD-bio were performed. Eight hundred of proteins were immunoprecipitated with anti-myc-magnetic beads and blotted using streptavidin conjugated with fluorophore (800 nm) and rabbit antibody (700 nm) to detect total PDI (rabbit-*anti*-PDI, Enzo, ADI-SPA-890).

PDI and actin sulfenylation were also assayed using recombinant rat PDI or purified actin (Cytoskeleton) as described by.^60^ Both proteins (5 μM) were reduced with TCEP 200 μM 15 min at room temperature, followed by filtration with BioSpin-P6 (Bio-Rad). Protein, either alone or co-incubated, were reacted with BTD-bio 100 μM in the absence or presence of H_2_O_2_ 100 μM during 1 h at 37°C. Detection of the sulfenylated fraction and total protein pool was performed as described above.

#### Measurement of reductase activity

Reductase activity assays were performed using purified mice aorta whole tissue lysate Briefly, thoracic and abdominal aorta were isolated from young and old mice, then lysed in Lysis buffer (HEPES 20 mM, 150 mM NaCl, EGTA 1 mM, MgCl_2_ 1.5 mM, 1% Triton X-, 10% glycerol) supplemented with complete protease inhibitors. Reductase activity was carried out by incubation of 40 μg aorta lysates in assay buffer (0.1 M potassium phosphate pH: 7.4, containing 2 mM EDTA), 5 μM DTT and 1 μM of di-eosin-GSSG. The increase in fluorescence was determined for 60 min by excitation at 520 nm and emission at 545 nm in a SpectraMax-M5. Reduction of 1 μM di-eosin-GSSG by 5 μM DTT served as a negative control. Three independent experiments (*n* = 5 each group: young and old) were performed, and each sample was detected in duplicate. The probe di-eosin-glutathione disulfide (GSSG) was prepared as previously described.^61^^,^^62^

#### Immunoprecipitation

Aortic homogenates from control C57BL/6 mice were lysed in RIPA buffer supplemented with iodoacetamide 10 mM, DTPA 100 μM, catalase 200 U/mL and protease inhibitors in the presence or absence of dimedone 5 mM. Two hundred and fifty micrograms of proteins were immunoprecipitated with rabbit-*anti*-PDI (Enzo). Western Blot detection in total lysate and immunoprecipitated fraction were performed in reducing or non-reducing conditions (as above) and blotted with mouse-β-actin (Sigma) antibody. Fluorescent detection was performed as described above.

#### Fluorescence microscopy

Cells or arteries were fixed with paraformaldehyde 4% - 30 min at room temperature (RT). Arteries were included in optimal cutting temperature compound and cryosections were performed at 10 μm thickness. Samples were permeabilized with Triton X-0.1% during 10 min (RT), blocked with bovine serum albumin (BSA 4%), incubated overnight with primary antibodies in BSA 1 mg/mL, followed by secondary fluorescent antibodies with phalloidin and Hoechst 33342 for nuclei or F-actin staining, respectively.

#### F-actin quantification

Actin filaments were submitted to distinct measurements including individual fiber thickness and cortical actin staining. Quantification of actin fiber thickness was performed using the open source plugin DiameterJ for ImageJ/Fiji.^63^ DiameterJ performs a two-step analysis in which images are segmented into a binary image and fibers are analyzed for their frequency, size, among other parameters. Cortical actin fluorescence was measured using ImageJ as described previously.^64^ Corrected total cell fluorescence (CTCF) was acquired in distinct regions of interest at the cortical area with the integrated density subtracted by the area of measurement multiplied by average image background.

#### Vascular reactivity

Aortic rings from rats were maintained in Krebs Henseleit buffer ((in mM) NaCl 130, KCl 4.7, KH_2_PO_4_ 1.18, MgSO_4_ 1.17, CaCl_2_ 1.16, glucose 5.5, NaHCO_3_ 14.9, and EDTA 0.026) stabilized at resting tension with 2 g or mice aortic rings with 0.5 g during 60 min changing Krebs Henseleit buffer every 20 min. Rings were constantly maintained with carbogenic mixture (O_2_ 95% and CO_2_ 5%) at 37°C. Independent receptor contraction was performed with KCl (20–100 mM) and receptor mediated contraction with phenylephrine 10^−9 - −4^ M. Tension was corrected by tissue dry weight.

#### Myogenic response

The animals were euthanized via isoflurane inhalation, and the entire intestine was immediately excised and maintained in a cold isolation solution containing (in mM) NaCl 118, KCl 5, NaH_2_PO_4_ 1.2, MgCl_2_ 1.2, CaCl_2_ 0.16, glucose 10, and HEPES 24 (pH 7.4 at room temperature). The explants were rinsed several times with the isolation solution, and branches of the third or fourth order originating from the superior mesenteric artery were dissected and cut into 2 mm segments. The segments were kept in the cold isolation solution until mounted with a transverse support of two 40 μm tungsten wires in a myograph (Danish Myo Technology - 620M). After the arteries were mounted, the isolation solution was replaced with physiological saline solution (PSS) containing (in mM) NaCl 119, KCl 4.7, KH_2_PO_4_ 1.18, MgSO_4_ 1.17, CaCl_2_ 1.6, glucose 5.5, NaHCO_3_ 25, and EGTA 0.03, continuously gassed with carbogen (95% O_2_ and 5% CO_2_). The preparations were not initially subjected to any tension, and the temperature was gradually increased to 37°C. A 30-min stabilization period was allowed before normalization. After stabilization, the normalization protocol was performed according to the manufacturer’s instructions (Myo Technology Normalization Guide - DMT), where the rings were stretched to a resting tension considered ideal for their internal diameter. For this, each artery was gradually stretched (30 μm) at 60-s intervals until a calculated transmural pressure of 100 mmHg was reached, followed by the release of the vessels to IC90, which is 90% of the internal circumference (IC100) at 100 mmHg. After each incremental force measurement, the corresponding transmural pressure was calculated using the Law of LaPlace via LabChart 8.0 software (DMT, Oxford, UK). From the distance between the wires (in mm), the length (in mm) of the vessels, internal circumference, transmural pressure, and vessel diameter were calculated, which was considered ideal up to a maximum of 200 μm. After the normalization protocol and a new stabilization period to record basal arterial activity, the evaluation of the vascular myogenic response under Dimedone action was initiated. A solution containing DMSO or Dimedone (1M – diluted in DMSO) was directly applied to each chamber of the organ bath containing 5 mL of PSS, resulting in a final concentration of 5 mM in each arterial ring. A 20-min period was allowed for the drug to take effect, after which the tension applied to each vessel was reset by bringing the wires back to their initial positions. Subsequently, the vessel stretching protocol was performed, in which each arterial segment was gradually stretched (30 μm) at 90-s intervals until again a calculated transmural pressure of 100 mmHg was reached, and the vessel responses to stretching were evaluated in each condition (treated with DMSO or Dimedone). The tension (mN/mm) was calculated from the recorded force and the length of each artery and the resting nominal pressure-diameter relationships from the calculated transmural pressures and the corresponding diameters of the vessels examined during stretching after incubation with DMSO or Dimedone.

#### Stretch-tension response

After active contraction studies (described above), arterial viscoelastic properties were assessed in rings maintained in Krebs solution without calcium and supplemented with EDTA 3 mM, sodium nitroprusside 100 μm and cytochalasin D 5 μM. Initial tension was maintained at around 0.1 g and stretched with 50 μm increment per step until vessel rupture. Tension values were corrected by dry weight as previously described.^41^

#### F-G-actin isolation

Separation of soluble (G) to filamentous (F) actin pools were performed using F/G-actin kit (Cytoskeleton) following manufacturer’s instructions. Arteries and isolated cells (5 x 10^5^) were immediately incubated in chilled lysis buffer (37°C) supplemented with ATP and protease inhibitor cocktail during 10 min. Arteries were lysed using mechanical disruption using ceramic beads under strong agitation (50 cycles/second) during 20 min. After, tissue debris were removed by gentle centrifugation (800 g–5 min, RT) and supernatant was submitted to ultracentrifugation (100.000 g, 60 min at 37°C) to isolate G-actin pool (supernatant) to F-actin fraction (pellet). F-actin was resuspended in urea 8 M using the same volume of the G-actin fraction. Actin was detected using β-actin or α-actin through Western Blot.

#### Local detection of hydrogen peroxide

One thousand of VSMC were transfected with plasmidial constructions (500 ng using lipofectamine) codifying Hyper-7 non-targeted (NT) or directed to F-actin (Hyper-actin). Twenty four hours after transfection, cells were serum starved during 1 h and maintained as control (DMSO) or treated with PDI inhibitors 16F16 3 μM or Bepristat 15 μM or Nox inhibitor GKT 40 μM during 30 min. Fluorescent images were acquired with Zeiss LSM 780 Confocal Microscope at CEFAP-USP using excitation at 400 and 500 nm for the protonated and deprotonated sensor, respectively, and emission at 510 nm. Plates were maintained under physiological conditions (temperature 37°C and CO_2_ 5%) and images were acquired before and after nocodazole 10 μM treatment during 5 min. Index of H_2_O_2_ production was calculated using “Ratio Plus” plug in from ImageJ, values were obtained using “Plot z axis profile” tool and expressed as ratio vs. time zero. The resulting image was converted into pseudocolor image using the “16-colors” lookup table ImageJ/Fiji tool as described.^58^

#### Total hydrogen peroxide detection

Amplex red assay coupled with horseradish peroxidase were performed to detect H_2_O_2_ in aortic rings as described previously.^65^ Aortic rings were incubated in Krebs-Hepes 20 mM containing amplex red 10 μM and horseradish peroxidase 1 U/ml. Vessels were maintained in control condition or stimulated with nocodazole 10 μM, phenylephrine 30 nM or, nocodazole plus phenylephrine, immediately after stimulation fluorescent detection was performed at 540 nm excitation and 590 nm emission during 60 min at 5 min interval.

### Quantification and statistical analysis

#### Statistical analysis

Data are presented as mean ± standard error and comparisons between two groups with *t* test when data follow normal distribution and Mann-Whitney test for nonnormal distribution. For three or more groups data compared with one way analysis of variance (ANOVA) with Bonferoni or Dunnett's post-test. Hyper ratio and vascular reactivity curves were compared using two-way ANOVA with Bonferoni post-test. All statistical differences were assumed when *p* < 0.05.
