# Supplementary material for: A mechanism for the disrupted redox regulation of vascular contractility during aging
Source: iScience. 2025 Nov 27;29(1):114264. doi: 10.1016/j.isci.2025.114264 (PMC12757538; doi:10.1016/j.isci.2025.114264)
Supplement: Document S1. Figures S1–S9 [file mmc1.pdf]

iScience, Volume *29*

Supplemental information

A mechanism for the disrupted redox regulation of vascular contractility during aging

Leonardo Y. Tanaka, Lucas F. Gutierre, Ricardo C. Massucatto, Geovana S. Garcia, Carolina M. Portas, Victor Debbas, Júlia M.F. de Souza, Tiphany C. De Bessa, Lívia Teixeira, Percíllia V.S. Oliveira, Beatriz P. Souza, Samantha K. Teixeira, Paola C. Branco, Ayumi A. Miyakawa, Renato S. Gaspar, Daniela Kajihara, Iuri C. Valadão, Amit Bhowmik, Kate Carroll, and Francisco R.M. Laurindo

Figures S1-S9

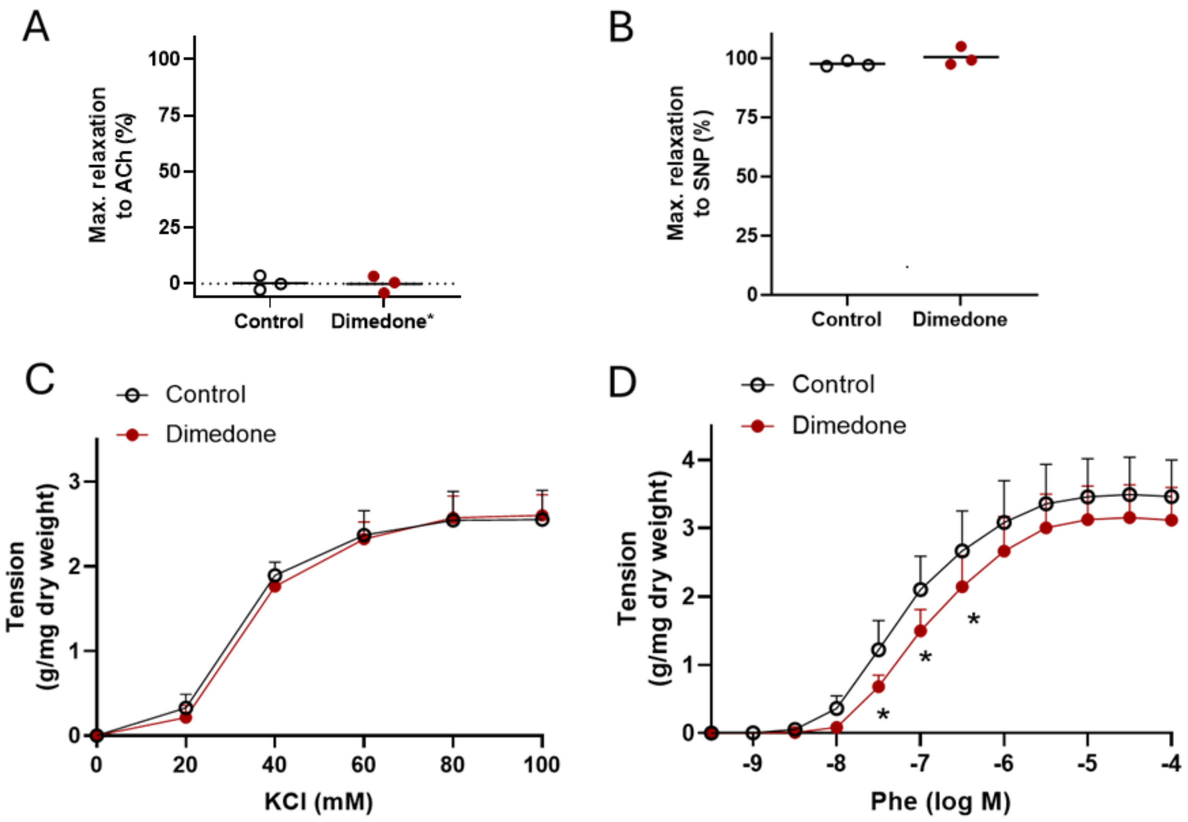

Figure S1. Effect of dimedone on the vascular reactivity of endothelium-denuded aorta, related to Figure 1. Endothelial layer of rat aortic rings was mechanically removed and conducted for vasomotor studies. (A) After contraction with phenylephrine (Phe, 10^{-6} M), aortic rings were relaxed with ACh 10^{-4} M and the maximum effect after 10 min was recorded. Dimedone* indicates the rings further used for dimedone 5 mM incubation. (B-D) Aortic rings treated with DMSO (Control) or dimedone 5 mM during 20 min were relaxed with sodium nitroprusside (SNP 10^{-4} M, B). SNP was added after stabilizing the maximum response in the phenylephrine curve depicted in "D". Vasoconstriction to KCl (20 – 100 mM, C) or Phe (10^{-9} - 10^{-4} M, D). *$p < 0.05$ Control. vs. Dimedone (two-way ANOVA followed by Bonferroni post-test). N=3 independent experiments. All graphs in this figure are presented as mean ± SEM.

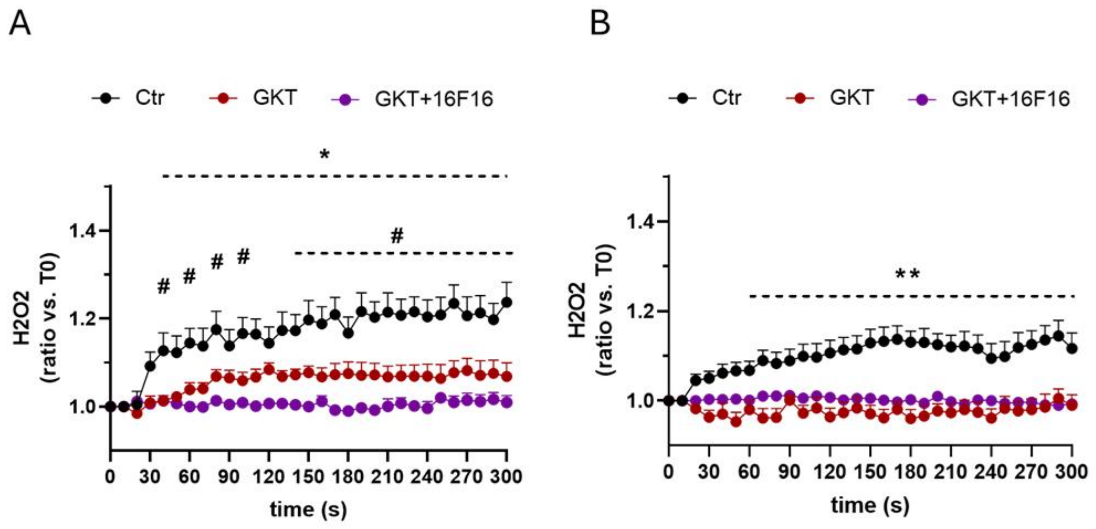

Figure S2. Combined effect of Nox and PDI inhibition on local H2O2 production during nocodazole stimulation, related to Figure 2. (A) A7R5 cells were transfected with H2O2 sensor Hyper-7 non-targeted (NT, A) or directed to F-actin (B) as described in Figure 1C. Twenty four hours after transfection cells were serum starved for 1 h and treated with DMSO or or Nox inhibitor GKT 40 μM alone or in combination with PDI inhibitor 16F16 3 μM for 30 min. Cells were treated with nocodazole 10 μM and fluorescence was measured at 400 nm or 500 nm excitation for detection of reduced or oxidized sensor, respectively. Ratio was calculated using image J/Fiji. Graphs depicts 5 min-time course of fluorescence ratio measured at distinct points at cortical region vs. time zero using NT-Hyper (A) or targeted to F-actin (B). *$p < 0.05$ Ctr vs. GKT + 16F16. $^{\#}p < 0.05$ Ctr vs. GKT. **$p < 0.05$ Ctr vs. other groups (both comparisons performed with two-way ANOVA followed by Bonferroni post-test). Measurements were performed in 5-10 cells from two independent experiments. Data are presented as mean ± SEM.

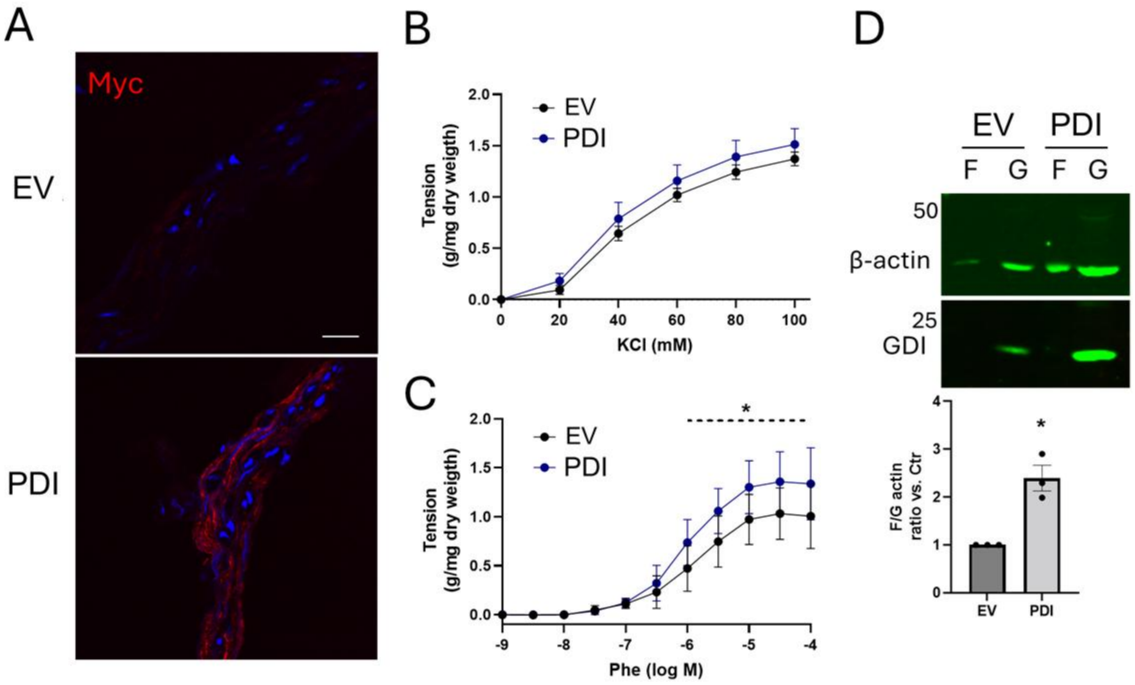

Figure S3. Effect of PDI overexpression on vascular contraction and F-actin assembly, related to Figures 2 and 3. Rat aortic rings were cultured for transfection with empty vector or cDNA coding rat PDI containing myc tag. Effects were measured 24 h after transfection. (A) PDI induction was measured through immunofluorescence for myc-tag (in red). Nuclei are shown in blue. Scale bar 20 μm. (B) Vascular contraction to KCl (20 – 100 mM), or (C) phenylephrine 10^{-9} – 10^{-4} M. *$p < 0.05$ vs. EV (two-way ANOVA followed by Bonferroni post-test) (D) Ratio between filamentous vs. globular β-actin. RhoGDIα depicts the purity of cytoskeleton fraction separation. *$p < 0.05$ vs. EV (paired *t*-test). Data are presented as mean ± SEM.

A

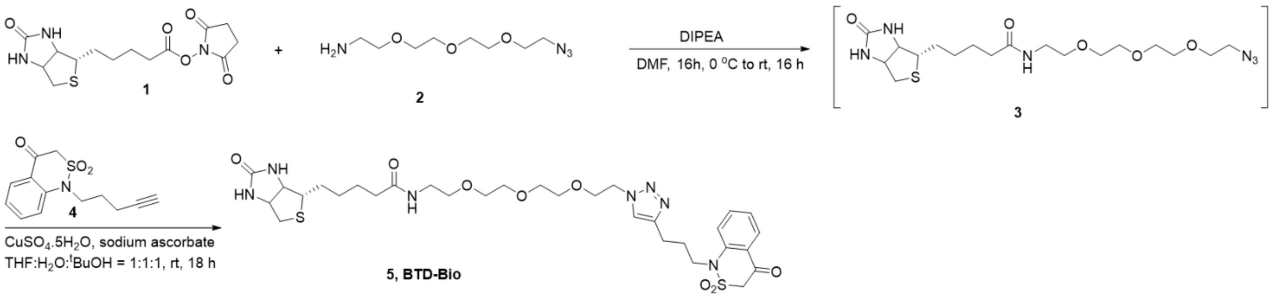
1
2
DIPEA
DMF, 16h, 0 °C to rt, 16 h
3
4
CuSO4.5H2O, sodium ascorbate
THF:H2O:tBuOH = 1:1:1, rt, 18 h
5, BTD-Bio

B

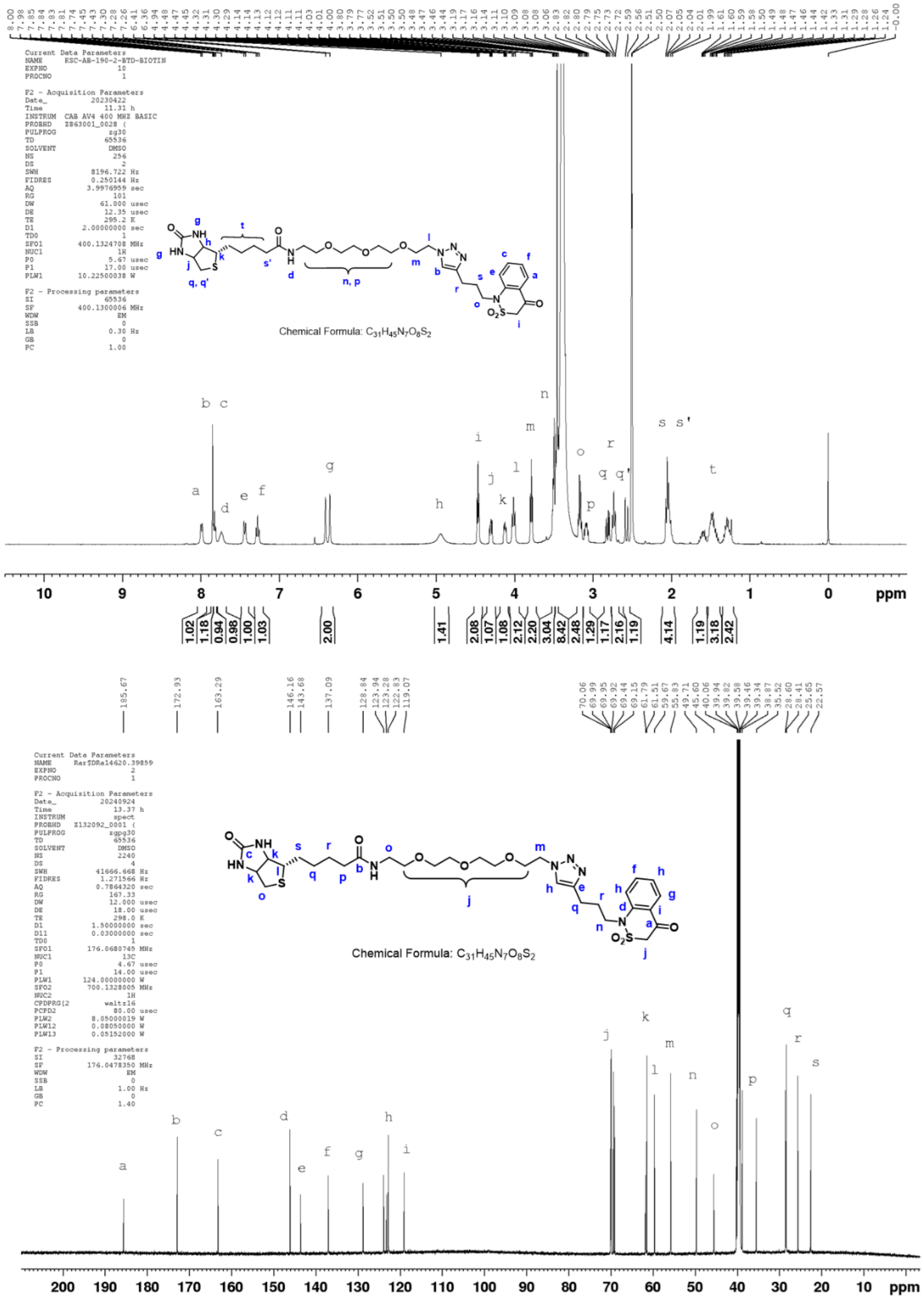
Chemical Formula: C31H45N7O8S2
ppm
Chemical Formula: C31H45N7O8S2
ppm

Figure S4. Synthesis of BTD-Bio, related to Star Methods. (A) N-(2-(2-(2-(2-(4-(3-(2,2-dioxido-4-oxo-3,4-dihydro-1H-benzo[c][1,2]thiazin-1-yl)propyl)-1H-1,2,3-triazol-1-yl)ethoxy)ethoxy)ethoxy)ethyl)-5-((4S)-2-oxohexahydro-1H-thieno[3,4-d]imidazol-4-yl)pentanamide (5 or BTD-Bio): To a solution of commercially available 1-amino11-azido-3,6,9-trioxaundecane (63 mg, 0.29 mmol) in DMF (2 mL) were added Biotin NHS (100 g, 0.29 mmol), and N,N-diisopropylethylamine (0.7 mL, 0.43 mmol, 1.5 equiv.) at 0 °C. After being heated to rt for 16 h, the reaction was evaporated under reduced pressure and filtered through a pad of silica (0-10 % methanol in CH_2Cl_2, 104 mg). LCMS analysis indicated the product was pure enough that could be used in the next step without further purification. To a solution of BTD **4** (61.6 mg, 0.234 mmol) in THF: H_2O: ^tBuOH = 1:1:1 (3 mL) was added Biotin-PEG3-N_3 **3** (104 mg, 0.234 mmol), $CuSO_4.5H_2O$ (29.2 mg, 0.117 mmol, 0.5 equiv.) and sodium ascorbate (15.4 mg, 0.078 mmol, 0.33 equiv.) at rt. After 18 h, the reaction mixture was evaporated under reduced pressure to give the crude mixture. The precipitate was by prep-HPLC purification (5-100% ACN in H_2O) to obtain the product **5** (90.6 mg, 0.128 mmol, 44% yield, 2 steps) as a pale greenish powder. ^1H NMR (DMSO-d_6, 400 MHz): 1.24-1.33 (m, 2H), 1.42-1.50 (m, 3H), 1.58-1.61 (m, 1H), 1.99-2.07 (m, 4H), 2.57 (d, J = 12.7 Hz, 1H), 2.73 (t, J = 7.5 Hz, 2H), 2.81 (dd, J = 12.7, 5.2 Hz, 1H), 3.06-3.19 (m, 1H), 3.17 (q, J = 5.9 Hz, 2H), 3.44-3.52 (m, 11H), 3.79 (t, J = 5.3 Hz, 2H), 4.01 (t, J = 7.5 Hz, 2H), 4.11-4.14 (m, 1H), 4.29-4.32 (m, 1H), 4.47 (t, J = 5.1 Hz, 2H), 4.94 (s, 1H), 6.36 (s, 1H), 6.41 (s, 1H), 7.28 (t, J = 7.3 Hz, 1H), 7.44 (d, J = 8.4 Hz, 1H), 7.74 (s, 1H), 7.81-7.85 (m, 2H), 7.99 (d, J = 7.8 Hz, 1H) ppm; (B) **^{13}C NMR** (DMSO-d_6, 175 MHz): 22.6, 25.6, 28.4, 28.6, 35.5, 45.6, 49.7, 55.8, 59.7, 61.5, 61.8, 69.1, 69.4, 69.92, 69.95, 69.99, 70.06, 119.1, 122.8, 123.3, 123.9, 128.8, 137.1, 143.7, 146.2, 163.3, 172.9, 185.7 ppm; ESI-LRMS calcd. for $[M+H]^+$ calcd. for $C_{31}H_{46}N_7O_8S_2$, 708.2849; found 708.2800.

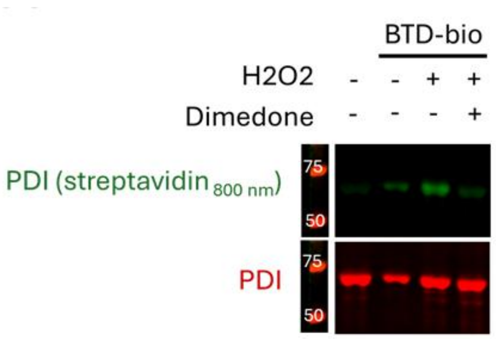

Figure S5. Effect of dimedone on PDI sulfenylation induced by H_2O_2, related to Figure 3. Pre-reduced recombinant rat-PDI 5 µM was incubated or not with BTD-bio 100 µM in the absence or presence of H_2O_2 100 µM during 1 h. Negative control was performed by blocking sulfenylated cysteines with dimedone 5 mM. PDI sulfenylation was detected with streptavidin conjugated with fluorophore (streptavidin 800 nm) and total PDI with anti-PDI. N=2 independent experiments.

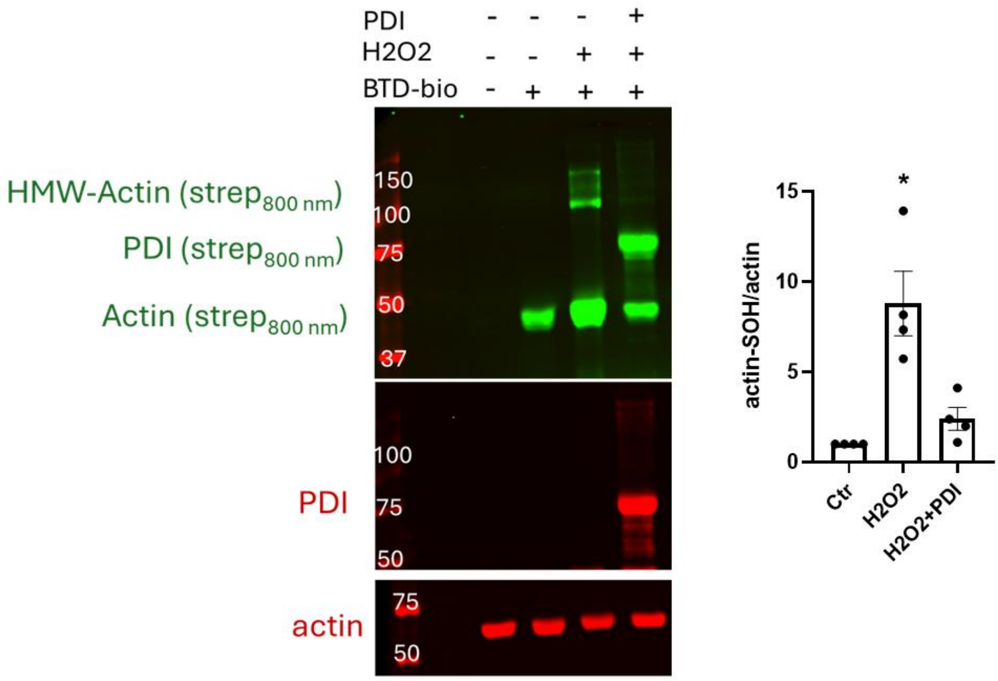

Figure S6. PDI decreases actin sulfenylation, related to Figure 3. Pre-reduced muscle actin 5 µM was incubated or not with BTD-bio 100 µM in the absence or presence of H_2O_2 100 µM or PDI 5 µM during 1 h. Actin and PDI sulfenylation were detected with streptavidin conjugated with fluorophore (strep 800 nm) and total actin or PDI with specific antibodies. *$p < 0.05$ vs. Ctr (one-way ANOVA followed by Dunnett´s post-test), n=4. Graph depicts mean ± SEM.

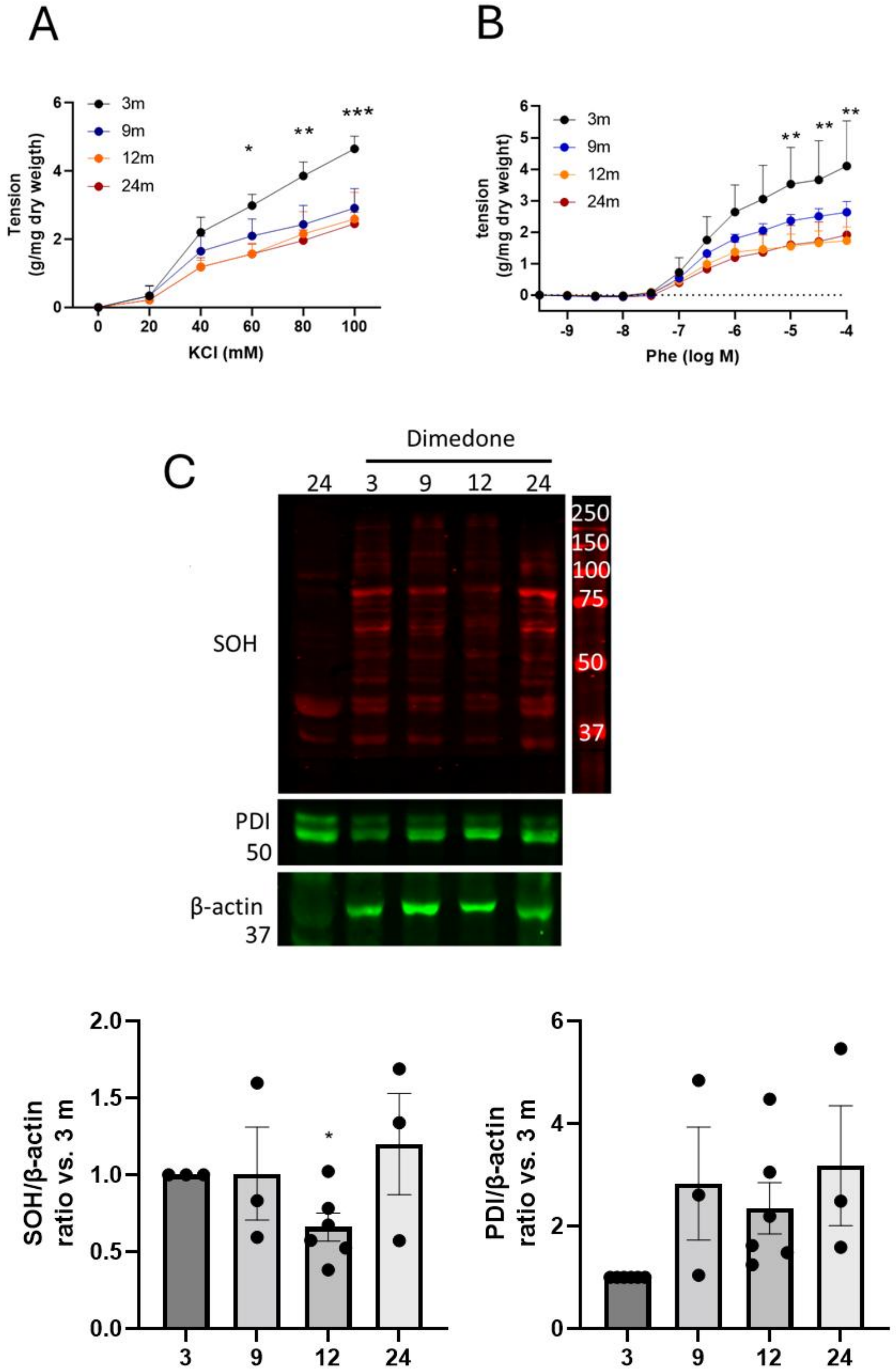

Figure S7. Effect of aging on vasoconstriction, protein sulfenylation and PDI expression, related to Figure 4. Vascular contraction to KCl (A) or phenylephrine (B) in aorta from C57Bl/6 mice mice at 3, 9, 12 or 24 months-age. *p < 0.05 3 m vs. 12 m, **p < 0.05 3 m vs. 12 and 24 m, ***p < 0.05 3 m vs. 9, 12 and 24 m (both comparisons performed with two-way ANOVA followed by Bonferroni post-test), n=4. (C) Aorta from C57BL/6 mice at 3, 9, 12 or 24 months old were lysed in buffer supplemented with dimedone 5 mM (additional supplements, see STAR Methods) for detecting sulfenylated (SOH) proteins. Representative images of SOH or PDI are shown (top). Graphs depict quantifications corrected by β-actin was used as loading control and expressed as ratio vs. 3 m (bottom). N=3-6. *p < 0.05 vs. 3 m vs. 12 m (one-way ANOVA followed by Dunnett´s post-test). All graphs in this figure are presented as mean ± SEM.

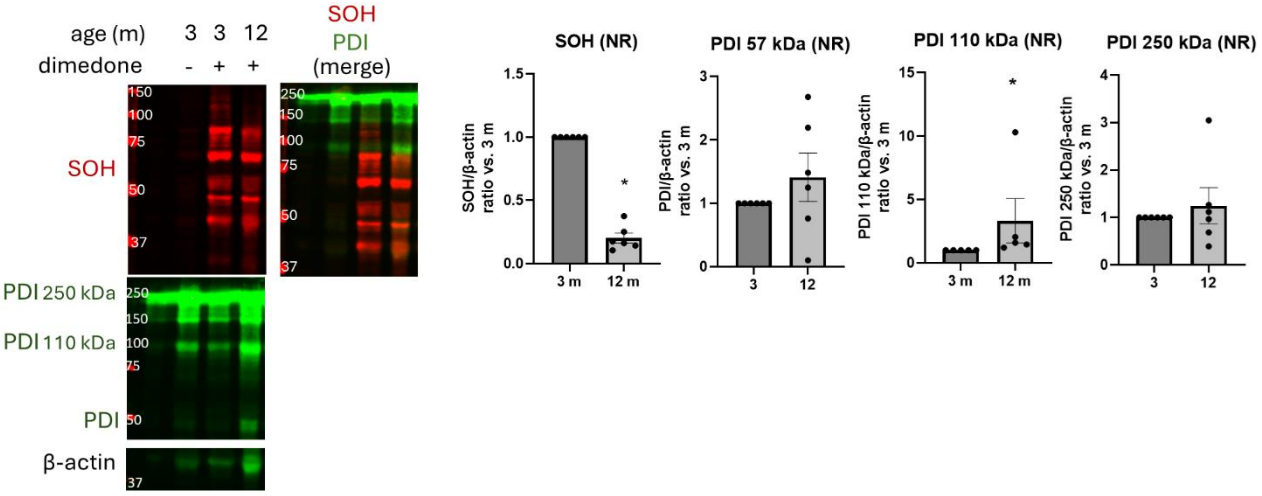

Figure S8. Western blot detection in non-reducing condition (NR) for protein sulfenylation (SOH), PDI or β-actin, related to Figure 4. PDI was depicited at distinct high molecular weight of around 110 and 250 kDa. Graphs at right depicting global SOH and PDI along with its redox complex were normalized by β-actin and expressed as ratio vs. 3 m. *$p < 0.05$ vs. 12 m (paired *t*-test), n=6. All graphs in this figure are presented as mean ± SEM.

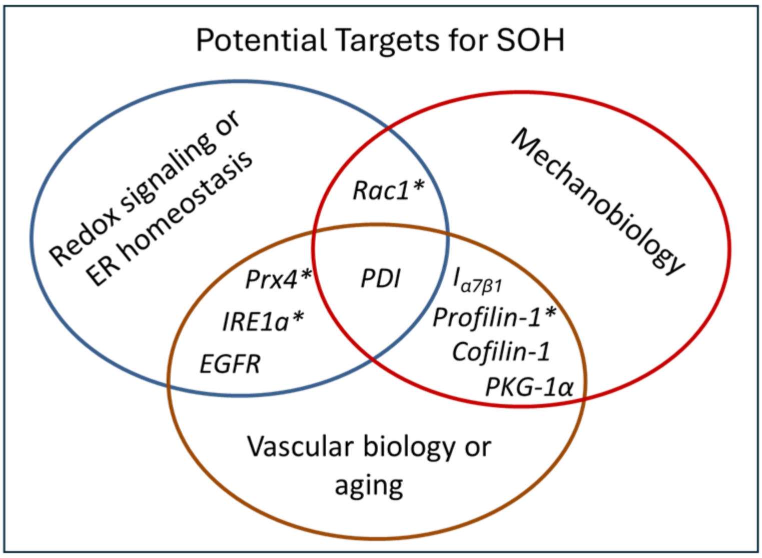

Figure S9. Potential protein targets for sulfenylation with additional relevance for the present study, related to "Discussion" section. *Depicts a known direct interaction with PDI.

1) Profilin-1 is an actin monomer binding protein which regulates actin polymerization by converting actin-ADP into ATP and allowing its incorporation in the actin filament. Sulfenylation of Profilin-1 has been detected in human colorectal carcinoma cell line [S1]. Through PDI substrate trapping approach (Moretti et al, unpublished data from our group), profilin-1 was pulled down with PDI. This experiment involved mutation of PDI resolving catalytic cysteines, thus, connecting PDI with targets for its reductase activity [S2]. Cofilin-1, an F-actin severing protein was reported to be sulfenylated in migrating breast cancer cells through a local increase of H_2O_2. Interestingly, sulfenylation of cysteines 139 and 147 leads to cofilin inhibition favoring actin assembly, which supports directional migration [S3].

2) Rac1, a small RhoGTPase, has the potential to be associated with important aspects related to the present study. First, it supports actin polymerization through WAVE-complex activation [S4], second it is a member of NADPH oxidase complex, such as the VSMC-relevant Nox1 [S5]. Third, it physically interacts with PDI in more aggressive cancer colon cells compared with less metastatic ones [S6]. Finally, Rac1 oxidant is targeted for sulfenylation [S7] and its oxidation increases its activity [S8].

3) Inositol-Requiring Enzyme 1 alpha (IRE1α), one of the proximal ER stress sensors presents differential downstream signaling after sulfenylation switching from unfolded protein response (UPR) signal to stress resistance activation and buffering aging phenotype [S9]. Interestingly, PDI phosphorylation was reported to affect its structure leading to holdase activity for IRE1alpha and attenuating excessive UPR activation [S10]. As UPR activation plays a role in VSMC senescence induction [S11], it is likely that increased PDI activity may overcome UPR signaling by indirect mechanisms associated with IRE1 or directly by assisting protein folding and thus attenuating aging effects.

4) Integrin α7β1, one of the heterodimeric receptor complexes from integrin family activated upon oxidation, more likely at the α7 protein, was reported to be sulfenylated during VSMC adhesion in laminin-111 by Nox4 activation [S12]. Evidence show that PDI oxidizes other integrins in vascular cells during mechanic stimulation [S13,S14]. Indeed, during VSMC repositioning by uniaxial stretch, a peri/epicellular pool of PDI, sustains local increase of sulfenylated proteins, balanced regulation of traction forces, actin fiber assembly and oxidation of Integrin β1 [S14].

5) Epidermal Growth Factor Receptor (EGFR), which was reported to be sulfenylated at Cys797 in human epidermoid carcinoma A431 cell line through Nox2 dependent activation [S15]. Interestingly, the tyrosine kinase activity of sufenylated EGFR is increased by such posttranslational modification and by inactivation of the tyrosine phosphatase Shp-2, which was also targeted by sulfenic acid upon EGFR activation [S15]. Importantly, vascular aging upregulates EGFR and its overactivation is involved with vascular inflammation [S16]. Another PDI family member, anterior gradient homolog 2 (AGR2) was reported to redox-interact with EGFR [S17].

6) Peroxiredoxin-4 is one of the top PDI-interactors pulled down by a redox-dependent substrate trapping approach [S18]. It was reported to be sulfenylated [S19] and to oxidize PDI during oxidative protein folding. Importantly, Prx4 counteracts atherosclerosis, decreasing plaque formation and features associated with plaque instability [S20], the latter also correlates with PDI inactivation by adduct formation with 4-hydroxynonenal [S21].

7) Protein kinase G Iα (PKG Iα), the final downstream target of NO signaling, was shown to be activated not only by increased cyclic GMP levels, but also by oxidation and homodimerization, leading to arterial relaxation [S22]. Recently, it was reported that sulfenylation of cysteine 42 plays a role on PKG Iα activation by physical exercise through ATP7A/SOD3 upregulating local peroxide level by exercise and restoring vasodilation in type 2 diabetes [S23].

Supplemental References

S1. Gupta, V., Yang, J., Liebler, D.C., and Carroll, K.S. (2017). Diverse Redoxome Reactivity Profiles of Carbon Nucleophiles. J Am Chem Soc 139, 5588-5595. 10.1021/jacs.7b01791.

S2. Stopa, J.D., Baker, K.M., Grover, S.P., Flaumenhaft, R., and Furie, B. (2017). Kinetic-based trapping by intervening sequence variants of the active sites of protein-disulfide isomerase identifies platelet protein substrates. J Biol Chem 292, 9063-9074. 10.1074/jbc.M116.771832.

S3. Cameron, J.M., Gabrielsen, M., Chim, Y.H., Munro, J., McGhee, E.J., Sumpton, D., Eaton, P., Anderson, K.I., Yin, H., and Olson, M.F. (2015). Polarized cell motility induces hydrogen peroxide to inhibit cofilin via cysteine oxidation. Curr Biol 25, 1520-1525. 10.1016/j.cub.2015.04.020.

S4. Lebensohn, A.M., and Kirschner, M.W. (2009). Activation of the WAVE complex by coincident signals controls actin assembly. Mol Cell 36, 512-524. 10.1016/j.molcel.2009.10.024.

S5. Cheng, G., Diebold, B.A., Hughes, Y., and Lambeth, J.D. (2006). Nox1-dependent reactive oxygen generation is regulated by Rac1. J Biol Chem 281, 17718-17726. 10.1074/jbc.M512751200.

S6. De Bessa, T.C., Pagano, A., Moretti, A.I.S., Oliveira, P.V.S., Mendonça, S.A., Kovacic, H., and Laurindo, F.R.M. (2019). Subverted regulation of Nox1 NADPH oxidase-dependent oxidant generation by protein disulfide isomerase A1 in colon carcinoma cells with overactivated KRas. Cell Death Dis 10, 143. 10.1038/s41419-019-1402-y.

S7. Sonego, G., Le, T.M., Crettaz, D., Abonnenc, M., Tissot, J.D., and Prudent, M. (2021). Sulfenylome analysis of pathogen-inactivated platelets reveals the presence of cysteine oxidation in integrin signaling pathway and cytoskeleton regulation. J Thromb Haemost 19, 233-247. 10.1111/jth.15121.

S8. Hobbs, G.A., Mitchell, L.E., Arrington, M.E., Gunawardena, H.P., DeCristo, M.J., Loeser, R.F., Chen, X., Cox, A.D., and Campbell, S.L. (2015). Redox regulation of Rac1 by thiol oxidation. Free Radic Biol Med 79, 237-250. 10.1016/j.freeradbiomed.2014.09.027.

S9. Hourihan, J.M., Moronetti Mazzeo, L.E., Fernández-Cárdenas, L.P., and Blackwell, T.K. (2016). Cysteine Sulfenylation Directs IRE-1 to Activate the SKN-1/Nrf2 Antioxidant Response. Mol Cell 63, 553-566. 10.1016/j.molcel.2016.07.019.

S10. Yu, J., Li, T., Liu, Y., Wang, X., Zhang, J., Shi, G., Lou, J., Wang, L., and Wang, C.C. (2020). Phosphorylation switches protein disulfide isomerase activity to maintain proteostasis and attenuate ER stress. Embo j 39, e103841. 10.15252/embj.2019103841.

S11. Wang, L., Wang, M., Niu, H., Zhi, Y., Li, S., He, X., Ren, Z., Wen, S., Wu, L., Zhang, R., et al. (2024). Cholesterol-induced HRD1 reduction accelerates vascular smooth muscle cell senescence via stimulation of endoplasmic reticulum stress-induced reactive oxygen species. J Mol Cell Cardiol 187, 51-64. 10.1016/j.yjmcc.2023.12.007.

S12. de Rezende, F.F., Martins Lima, A., Niland, S., Wittig, I., Heide, H., Schroder, K., and Eble, J.A. (2012). Integrin alpha7beta1 is a redox-regulated target of hydrogen peroxide in vascular smooth muscle cell adhesion. Free Radic Biol Med 53, 521-531. 10.1016/j.freeradbiomed.2012.05.032.

S13. Araujo, T.L., Zeidler, J.D., Oliveira, P.V., Dias, M.H., Armelin, H.A., and Laurindo, F.R. (2017). Protein disulfide isomerase externalization in endothelial cells follows classical and unconventional routes. Free Radic Biol Med 103, 199-208. 10.1016/j.freeradbiomed.2016.12.021.

S14. Tanaka, L.Y., Araujo, T.L.S., Rodriguez, A.I., Ferraz, M.S., Pelegati, V.B., Morais, M.C.C., Dos Santos, A.M., Cesar, C.L., Ramos, A.F., Alencar, A.M., and Laurindo, F.R. (2018). Peri/epicellular Protein Disulfide Isomerase-A1 Acts as an Upstream Organizer of Cytoskeletal Mechanoadaptation in Vascular Smooth Muscle Cells. Am J Physiol Heart Circ Physiol. 10.1152/ajpheart.00379.2018.

S15. Paulsen, C.E., Truong, T.H., Garcia, F.J., Homann, A., Gupta, V., Leonard, S.E., and Carroll, K.S. (2011). Peroxide-dependent sulfenylation of the EGFR catalytic site enhances kinase activity. Nat Chem Biol 8, 57-64. 10.1038/nchembio.736.

S16. Krug, A.W., Allenhöfer, L., Monticone, R., Spinetti, G., Gekle, M., Wang, M., and Lakatta, E.G. (2010). Elevated mineralocorticoid receptor activity in aged rat vascular smooth muscle cells promotes a proinflammatory phenotype via extracellular signal-regulated kinase 1/2 mitogen-activated protein kinase and epidermal growth factor receptor-dependent pathways. Hypertension 55, 1476-1483. 10.1161/hypertensionaha.109.148783.

S17. Dong, A., Wodziak, D., and Lowe, A.W. (2015). Epidermal growth factor receptor (EGFR) signaling requires a specific endoplasmic reticulum thioredoxin for the post-translational control of receptor presentation to the cell surface. J Biol Chem 290, 8016-8027. 10.1074/jbc.M114.623207.

S18. Zito, E., Melo, E.P., Yang, Y., Wahlander, Å., Neubert, T.A., and Ron, D. (2010). Oxidative protein folding by an endoplasmic reticulum-localized peroxiredoxin. Mol Cell 40, 787-797. 10.1016/j.molcel.2010.11.010.

S19. Zhu, L., Yang, K., Wang, X., and Wang, C.C. (2014). A novel reaction of peroxiredoxin 4 towards substrates in oxidative protein folding. PLoS One 9, e105529. 10.1371/journal.pone.0105529.

S20. Guo, X., Yamada, S., Tanimoto, A., Ding, Y., Wang, K.Y., Shimajiri, S., Murata, Y., Kimura, S., Tasaki, T., Nabeshima, A., et al. (2012). Overexpression of peroxiredoxin 4 attenuates atherosclerosis in apolipoprotein E knockout mice. Antioxid Redox Signal 17, 1362-1375. 10.1089/ars.2012.4549.

S21. Muller, C., Bandemer, J., Vindis, C., Camare, C., Mucher, E., Gueraud, F., Larroque-Cardoso, P., Bernis, C., Auge, N., Salvayre, R., and Negre-Salvayre, A. (2013). Protein disulfide isomerase modification and inhibition contribute to ER stress and apoptosis induced by oxidized low density lipoproteins. Antioxid Redox Signal 18, 731-742. 10.1089/ars.2012.4577.

S22. Burgoyne, J.R., Madhani, M., Cuello, F., Charles, R.L., Brennan, J.P., Schroder, E., Browning, D.D., and Eaton, P. (2007). Cysteine redox sensor in PKGIa enables oxidant-induced activation. Science 317, 1393-1397. 10.1126/science.1144318.

S23. Sudhahar, V., Abdelsaid, K., Eaton, P., Kelley, S., Ushio-Fukai, M., and Fukai, T. (2025). Exercise-Induced SOD3/H(2)O(2) Sulfenylates PKG1α to Restore Vasodilation in Diabetes. Circ Res 137, 449-452. 10.1161/circresaha.125.326586.
